# Continuous goal representations: Distance in representational space affects goal switching

**DOI:** 10.3758/s13421-024-01675-9

**Published:** 2025-01-21

**Authors:** Ulrike Senftleben, Simon Frisch, Maja Dshemuchadse, Stefan Scherbaum, Caroline Surrey

**Affiliations:** 1 Department of Psychology, Technische Universität Dresden, Zellescher Weg 17, 01062 Dresden, Germany; 2 Faculty of Social Sciences, Zittau-Görlitz University of Applied Science, Theodor-Körner-Allee 16, 02763 Zittau, Germany

**Keywords:** Goal representations, Continuity of mind, Set shifting, Cognitive control

## Abstract

Theorists across all fields of psychology consider goals crucial for human action control. Still, the question of how precisely goals are represented in the cognitive system is rarely addressed. Here, we explore the idea that goals are represented as distributed patterns of activation that coexist within continuous mental spaces. In doing so, we discuss and extend popular models of cognitive control and goal-directed behavior, which implicitly convey an image of goals as discrete representational units. To differentiate empirically between discrete and continuous formats of goal representation, we employed a set-shifting paradigm in which participants switched between color goals that varied systematically in their distance in representational space. Across three experiments, we found that previous goals biased behavior during goal switches and that the extent of this bias decreased gradually with the previous goal’s distance in color space from color information in the current trial. These graded effects of goal distance on performance are difficult to reconcile with the assumption that goals are discrete representational entities. Instead, they suggest that goals are represented as distributed, partly overlapping patterns of activation within continuous mental spaces. Moreover, the monotonous effects of distance in representational space on performance observed across all conditions in all experiments imply that the spreading of goal activation in representational space follows a monotonous (e.g., bell-shaped) distribution and not a nonmonotonous (e.g., Mexican-hat shaped) one. Our findings ask for a stronger consideration of the continuity of goal representations in models and investigations of goal-directed behavior.

**Supplementary information:**

The online version contains supplementary material available at 10.3758/s13421-024-01675-9.

The ability to act voluntarily and align one’s actions with one’s goals is considered the pinnacle of human cognition. Consequently, goals are integral parts of many influential theories in psychology whose theoretical and empirical value has been richly proven (e.g., Baltes & Baltes, 1990; Bandura, 1986; Carver & Scheier, 2001; Deci & Ryan, 2000; Locke & Latham, 2002; Miller, 2000). Yet it is important to note that the literature lacks a clear definition of the umbrella term *goal*. While in some cognitive approaches goals are defined as mental sets (Monsell, 2017), in other lines of research, goals are attentional templates (Geng et al., 2017) or task rules (Meiran & Kessler, 2008) or control representations (Cohen, 2017; Cohen et al., 1990). Given the importance of goals in psychological research, it is surprising how little consensus there is about how goals are best conceptualized (see Austin & Vancouver, 1996; Elliot & Fryer, 2008). This lack of clarity is not just noticeable in verbal theories of volition and motivation. It also holds for computational models of cognitive control (e.g., Botvinick et al., 2001; Cohen et al., 1990; Frisch et al., 2015; Gilbert & Shallice, 2002; Scherbaum et al., 2012; Shenhav et al., 2013; Verguts & Notebaert, 2008), which arguably provide the most explicit characterization of goal-directed behavior. These models, too, appear to treat the question of what goals are and how they are represented in the cognitive system more as an afterthought. By implementing goal representations as singular neural network nodes that link singular stimuli to singular responses, they propagate an image of goal representations that resembles the idea of “grandmother cells”—that is, of singular neurons that represent complex objects (such as one’s grandmother). While this hypothesis has been vocally criticized in the neuroscience literature and has been largely succeeded by the assumption that information is represented by distributed patterns of activation of larger populations of neurons in the human cortex (Kiefer & Pulvermüller, 2012; Xue, 2018; but see Bowers, 2017), the image of singular, discrete goal representations prevails in research on volition and cognitive control (e.g., Dietrich & Markman, 2003; Markman & Brendl, 2000). In the present article, we examine this implicit assumption and explore whether goals are distributed representations as well. Since there is not a clear definition of goals in the cognitive control literature, we decided to focus on the continuous expressions of goals at the perceptual level by investigating color goals. Hence, we conceptualize goals as higher-level representations influencing lower-level processes. To this end, we report a series of three set-shifting experiments in which participants switched between color goals that differed systematically in their level of similarity. If goals were represented as distributed patterns of activation, goal switching performance should vary as a function of goal-to-goal similarity because the level of overlap between consecutive goal representations would vary as well. In contrast, if goals were best characterized as discrete representational units, goal similarity should not affect switching performance. In the following, we elucidate the background of our research by, first, elaborating on the discrete conception of goals in cognitive control research, second, contrasting this perspective with more recent theories and findings suggesting a distributed format of mental representation and, third, presenting our research rationale and differential predictions to distinguish the two. Furthermore, after providing initial evidence for our continuous goal hypothesis in Experiment 1, we will address the question how precisely goal activation may spread through representational space.

## Goals as discrete representations

Cognitive control describes the ability to process goal-relevant information in the face of interference (e.g., Miller, 2000): Even though our surroundings are filled with an abundance of distractions, merely thinking about a goal (e.g., to meet a friend in a crowded bar) reliably draws attention toward objects associated with this goal (e.g., the bright green jacket she usually wears) and activates appropriate responses (e.g., tapping on our friend’s shoulder and saying “Hi!”). Over the past decades, our understanding of such acts of control has been greatly improved by a number of influential computational models that aim to provide mechanistic explanations for controlled behavior (Botvinick et al., 2001; Cohen et al., 1990; Frisch et al., 2015; Gilbert & Shallice, 2002; Scherbaum et al., 2012; Shenhav et al., 2013; Verguts & Notebaert, 2008). A feature shared by all these models is that they rely on higher-order goal representations to implement control. These goal units, which are at times also referred to as attentional, task demand, or context representations, bias processing in lower (i.e., sensory and motor) levels of the cognitive system such that task-relevant information gains stronger influence on response selection than task-irrelevant information. This way, goal representations resolve interference and ensure proper responding in situations where conflicting stimulus features call for incompatible responses or where habitual responses must be overcome by new response mappings to complete a task.

Goal representations are thus crucial for understanding willful behavior. However, the focus of most current models of cognitive control lies not so much on the question of what goals are and how they are represented in the cognitive system, but rather on how goals are updated or shielded in response to changing task demands. Implicitly, most models of cognitive control convey an image of goals as discrete, static entities: Goals are modelled as single units within neural networks which represent specific task-relevant sources of information or single stimulus features that are available in the environment (Botvinick et al., 2001; Cohen et al., 1990; Gilbert & Shallice, 2002; Shenhav et al., 2013; Verguts & Notebaert, 2008). Apart from inhibitory connections which ensure that only one goal is active (i.e., only one stimulus feature lies in the focus of attention) at any point in time, the different units in the goal layer largely operate independently from each other. This take on goals can explain important findings in the cognitive control literature with low computational effort (see Botvinick & Cohen, 2014, for an overview). However, in the real world, goal-relevant and goal-irrelevant sources of information usually are not perfectly distinguishable. They often lie on different points of the same physical dimension by differing in their location, size, pitch, or color, and experience tells us that our ability to respond correctly often depends on how distinct task-relevant and distracting information are regarding this dimension. Searching for your friend’s green jacket in a bar is quite easy at your regular after-hour party where most attendees wear muted colors. On St. Patrick’s Day, however, this task becomes much more challenging due to the various shades of green you are surrounded with. Standard models of cognitive control leave aside this natural gradedness, and this disregard is also reflected in the way goal-directed behavior is studied in the lab. Researchers usually rely on established, discrete paradigms in which participants respond to discrete classes of stimuli by giving discrete responses. For example, when researchers conduct task- or set-switching experiments to investigate how the cognitive system adapts to changing task demands (e.g., Dreisbach & Goschke, 2004; Frisch et al., 2015; Longman et al., 2013), they usually have participants alternate between a small, limited number of discrete goals (e.g., respond to either red, green, or blue stimuli; attend to either the magnitude or the parity of a digit) and give discrete answers (e.g., pressing left or right buttons). Hence, for many models, implementing goals as discrete and static might not reflect a theoretical assumption of how goals are represented, but might rather simply reflect the nature of the tasks that these models are trying to explain. However, by relying on such discretized paradigms and models, research in the field overlooks important alternative perspectives on goal representations.

## Goals as continuous representations

**Fig. 1:**
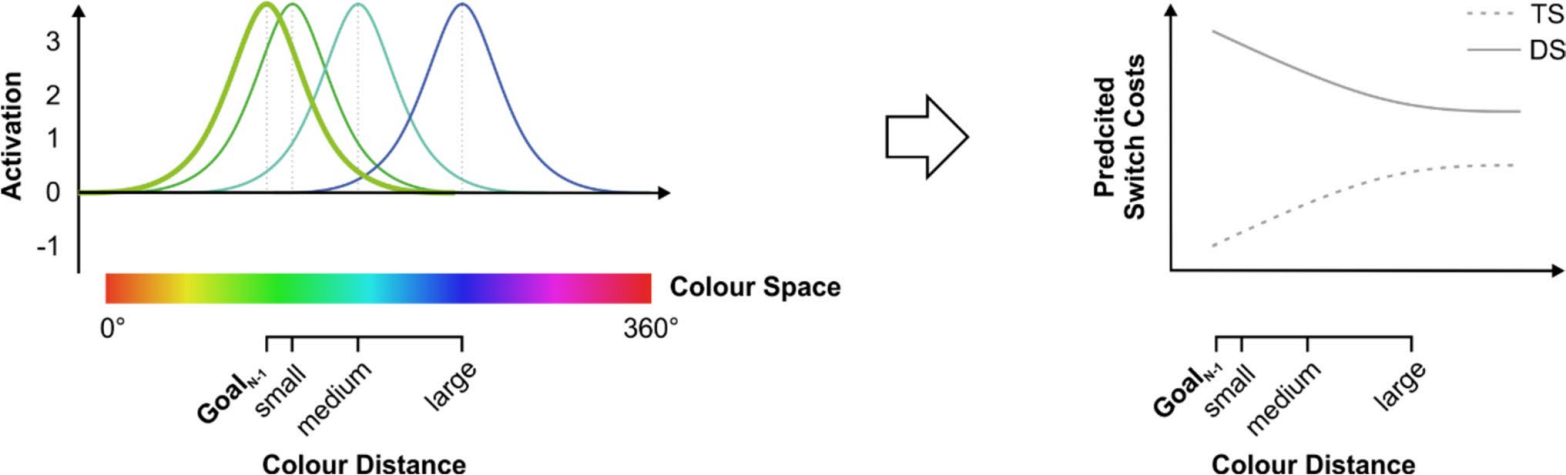
**Left:** Illustration of continuous color goal representations. A goal is not defined by a singular color value but by a distributed pattern of activation within continuous colors space, which is centered on the to-be-coded feature value. Accordingly, different goal representations overlap to varying degrees, depending on their distance in color space. **Right:** Predicted performance costs (e.g., response times, error rates) for goal switches of varying distance in color space in the target-similar (TS) and the distracter-similar (DS) conditions of our set-shifting paradigm (see below for details). Under the assumption that goal representations follow a monotonic (here: bell-shaped) activation function, performance during switches in the TS condition should decline continuously with increasing distance between the previous color goal and current target color. In contrast, during switches in the DS condition, performance should improve continuously with increasing distance between previous color goal and current distracter color. (Color figure online)

Recently, more dynamic approaches to cognition (e.g., Kiyonaga & Egner, 2016; Lins & Schöner, 2014; Oberauer & Lin, 2017; Sandamirskaya et al., 2013; Scherbaum et al., 2008; Spivey & Dale, 2004; Wimmer et al., 2014) provide evidence that are not entirely consistent with the discrete conception of goal representations and cognitive control. Building on the assumption that thought and action are grounded in the physical world (e.g., Barsalou, 2008), they assume that the cognitive system represents information as patterns of neural activation that unfold within continuous mental spaces. The idea of a spatially continuous format of mental representation builds on the well-established findings of population coding (e.g., Georgopoulos et al., 1986), neural tuning curves (e.g., Treue, 2001), and bump attractor dynamics (e.g., Wimmer et al., 2014) in the brain. Single neurons in the sensory cortex are only roughly tuned to specific values of physical stimulus features like pitch, tilt or color. Therefore, their activation alone cannot explain the high level of precision with which information is stored in our brains. Instead, it is the characteristic pattern of activation produced by the joint firing of large populations of neurons that serves as the fundamental unit of mental representation, a principle that is commonly referred to as population vector coding (see Lins & Schöner, 2014; Sandamirskaya et al., 2013). Accordingly, within this framework, units of information are modelled as peaks or “bumps” (Wimmer et al., 2014) of activation that are defined over hypothetical, topologically ordered mental spaces comprising all neural units that code a given sensory dimension such as pitch, tilt, or color (see Fig. 1). The bumpy shape emerges because activating a goal representation (e.g., spotting your friend’s green jacket) not only causes units that are fine-tuned to the physical value of interest to fire at a high rate (e.g., the specific shade of green the jacket is made of), but also results in the firing of units that are tuned to slightly different colors (e.g., a “warmer” olive-green that blends into yellow or a “cooler” forest green that blends into turquoise), albeit to a lesser degree. Thus, instead of reducing mental representations to discrete, singular entities that exist independently from each other, these approaches stress that the mind is grounded in a plethora of continuous representational spaces. These continuous representational spaces are spanned by the features of the physical world within which percepts, concepts, and goals are represented and coexist as graded, distributed patterns of activation (Johnson et al., 2008; Lins & Schöner, 2014; Sandamirskaya et al., 2013).

If goal information was indeed represented in such a continuous fashion, one would expect to find traces of this representational format in human goal-directed behavior. In particular, a continuous perspective on goals would predict that the level of interference between competing or successive goal representations should vary systematically with the level of goal overlap in representational space. While research exploring this prediction is currently lacking from the cognitive control literature, early work by Duncan and Humphreys (1989) on visual attention showed that visual search performance is affected by target–distractor similarity and that this holds true for different types of visual stimuli and dimensions, such as color, tilt or shape. For example, detecting a red target amongst pink distractors, where target–distractor similarity is high, was found to be more difficult than detecting a red target amongst green distractors. In addition, a small number of studies on the dynamics of working memory—the functional unit in which goal representations are instantiated, maintained, and updated (e.g., Gruber & Goschke, 2004; Miller & Cohen, 2001)—suggests that it may provide an adequate description of how goals are represented. For example, it has been shown that when subjects hold multiple locations (Spencer & Hund, 2003) or colors (Johnson et al., 2008, 2009a, 2009b) in working memory in parallel, these representations are drawn toward each other and can even merge if they are close enough in representational space. Similarly, several studies have demonstrated that short-term memories of stimulus features like tilts (Rademaker et al., 2015) or locations (Guérard & Tremblay, 2011; Johnson & Spencer, 2016; Van der Stigchel et al., 2007) are being “pulled” toward the tilts or locations of task-irrelevant stimuli presented during the retention interval. Such systematic effects of stimulus similarity on working memory performance are incompatible with classic models of working memory that represent information in independent slots and chunks (e.g., Luck & Vogel, 1997). Instead, they suggest that working memory relies on an analogue format for representation that results in interference when items are close or similar to each other with regard to their task-relevant features (Johnson & Spencer, 2016; Kiyonaga & Egner, 2016; Wimmer et al., 2014).

## The present study

The purpose of the present research is to test this continuous perspective within the realm of goal-directed behavior. To this end, we conducted three set-shifting experiments that shared a similar design. Participants responded to one of two differently colored digits that appeared in a cued target color (e.g., red) while ignoring a second, task-irrelevant digit that was presented in a different distracter color (e.g., blue). Colors switched repeatedly over the course of the experiments. The crucial manipulation in each of the experiments was that target and distracter colors after the switch differed systematically in their distance in color space relative to the target color prior to the switch. In the *target-similar* condition, the new *target* color was either identical or similar to the previous target color (e.g., red) to varying degrees (red vs. orange vs. yellow) while the distracter stimuli appeared in an unrelated color (e.g., green). In contrast, in the *distracter-similar* condition, the new *distracter* color was either identical or similar (e.g., red vs. orange vs. yellow) to the previous target color (e.g., red) while target stimuli appeared in an unrelated color (e.g., green, see the Methods section of Experiment 1 for a detailed description of the paradigm).

The assumption that goal representations are continuous in representational space leads to specific predictions of how switch costs—the relative decrease in performance when a goal is switched as compared to when it is repeated (see Kiesel et al., 2010)—are modulated by goal distance in representational space. First, in the target-similar condition, performance should be best (i.e., response times and error rates should be lowest) when previous and current task goal are identical because activation patterns in representational space overlap perfectly and no redistribution of activation is necessary. However, the larger the dissimilarity between consecutive goal representations becomes, the less pronounced this benefit should be as the overlap in goal activation decreases (see Fig. 1). Second, in the distracter-similar condition, we would expect an inversed behavioral pattern: While a high overlap between previous target and current distracter colors should incur large costs on behavior due to the distraction induced by persisting goal activation (e.g., Dreisbach & Goschke, 2004; Frisch et al., 2015; Goschke, 2000; Longman et al., 2013), the size of this effect should decrease the more dissimilar the new distracter color is relative to the previous target color (see Fig. 1).

Taken together, the core prediction following from a continuous perspective on goals regarding our paradigm is that the effect of persisting goal activation on behavior during switch trials should vary gradually as a function of goal-to-goal or goal-to-distracter similarity in the two switching conditions. Importantly, such graded effects of distance in representational space are specific for the assumption that goals are represented continuously in representational space and that they overlap with respect to their similarity. Hence, these graded effects would be incompatible with the implicit assumption of standard models of cognitive control (e.g., Botvinick et al., 2001; Gilbert & Shallice, 2002; Shenhav et al., 2013; Verguts & Notebaert, 2008) that goals are isolated entities that do not overlap. Under this assumption, goal switching should be equally difficult, irrespective of goal-to-goal distance in representational space.

## Experiment 1

Experiment 1 laid the foundation for a detailed investigation of the continuity of goal representations by implementing a novel set-shifting paradigm that systematically varied the level of color similarity between previously and currently task-relevant and task-irrelevant color information in representational space. If goal representations were continuous in representational space (e.g., Johnson et al., 2009a, 2009b; Sandamirskaya et al., 2013; Wimmer et al., 2014), we would expect a graded effect of color distance on performance: While performance in switch trials of the target-similar (TS) condition should decrease with color distance, it should increase in switch trials of the distracter-similar (DS) condition.

### Methods

#### Participants

Twenty-five participants^1^ (19 women, *M*_Age_ = 20.96 years) from Technische Universität Dresden took part in the experiment and received 7€ or course credits for their participation. All participants had normal or corrected-to-normal vision and color vision. The study was approved by the ethics committee of the Technische Universität Dresden, and all participants gave informed consent in accordance with the Declaration of Helsinki.

#### Apparatus and stimuli

**Fig. 2:**
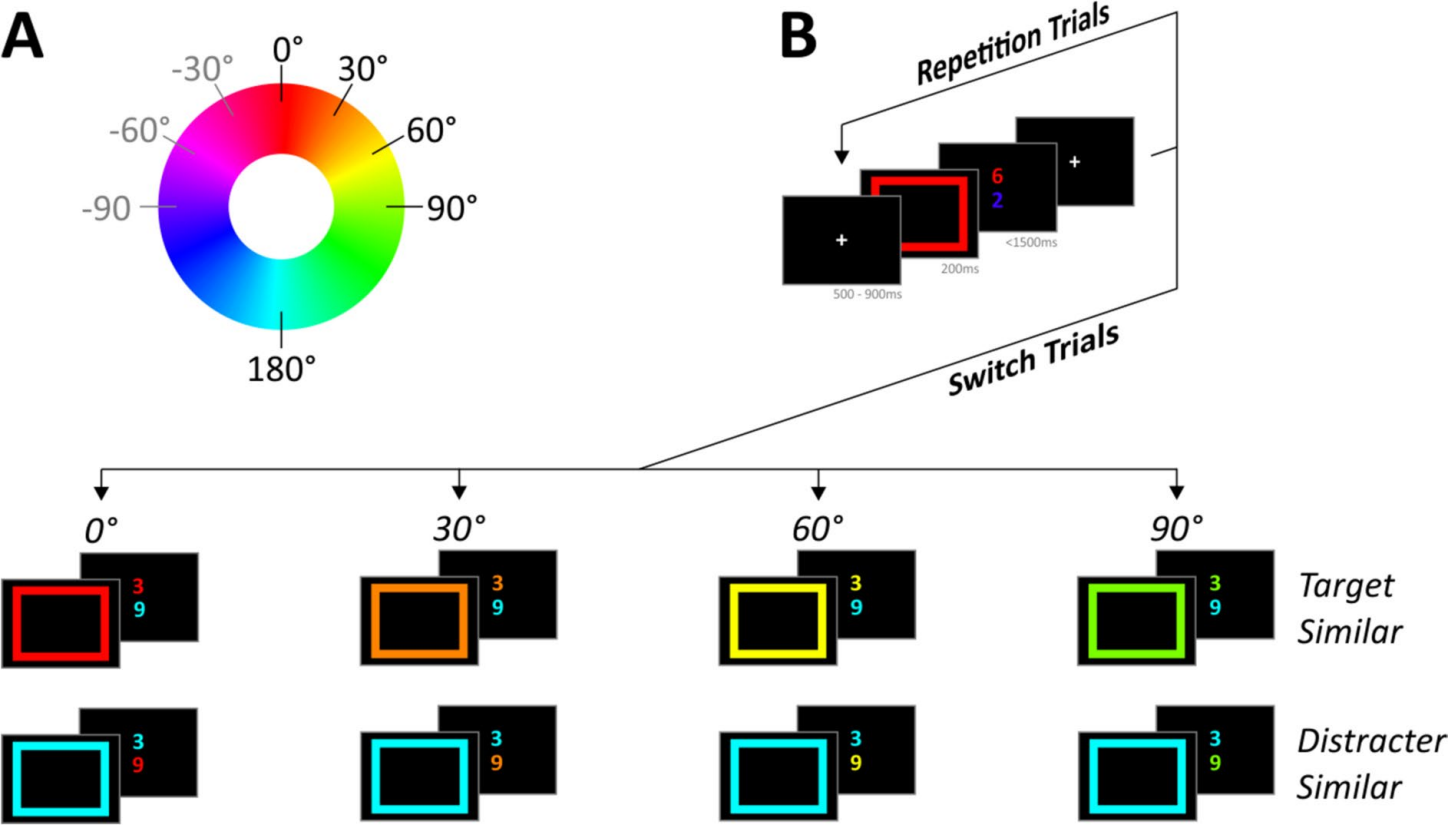
**A** Color circle illustrating the HSV color space (used throughout all experiments) and switch distances (used in Experiment 1). Note that the seed color (marked with 0° in this figure) represents the previous target color and thus changed every switch. **B** Procedure of Experiment 1. Participants had to indicate whether the digit appearing in the color indicated by the rectangular color cue was smaller or larger than 5. After 6 to 8 repetition trials, target and distracter colors switched. In the target-similar condition, the new target color was similar to the previous target color. In the distracter-similar condition, the new distracter color was similar to the previous target color. The distance in color space was varied in steps of 30° between 0° and 90°. Note that the location of target vs. distracter digits (upper vs. lower) was balanced across trials and that the color of the second digit changed as well from trial to trial (see main text for details on color selection). (Color figure online)

Participants were seated approximately 60 cm away from a 17-in. computer screen operating on a resolution of 1,280 × 1,024 pixels. A 90 × 90 pixel white fixation cross served as a fixation cross at the center of the screen. Rectangular frames of different colors with a size of 210 × 210 pixels and a line width of 20 pixels served as cues. The digits 1 to 9 (except 5), sized 80 × 80 pixels, served as target and distracter stimuli and appeared 15 pixels above and below the fixation cross’s outer margins (1.24° of visual angle from the center of the screen). This setup ensured that cue, target, and distracter stimuli did not overlap spatially. Hues for cues, target, and distracter stimuli were selected in steps of 30° relative to a randomly chosen starting point from a circular hue-saturation-value (HSV) color space (see Fig. 2A). Saturation and value were held equal (maximal) for all stimuli. The Psychophysics Toolbox 3 (Brainard, 1997; Pelli, 1997) in MATLAB 2010 (The MathWorks, Inc.) on a Windows PC was used for presentation.

#### Procedure

Each trial consisted of three phases. First, a fixation cross was presented for a randomly chosen intertrial interval of 500 ms, 700 ms, or 900 ms. Second, a rectangular frame cueing the relevant target color appeared for 200 ms. Third, the target and distracter digits appeared above and below the center of the screen, with the location (upper vs. lower) of target and distracter digits being balanced across trials. Subjects were instructed to respond to the magnitude (< 5 vs. > 5) of the digit appearing in the cued target color without paying attention to the second, task-irrelevant digit by pressing a left (< 5) vs. right (> 5) key on a standard keyboard. The next trial started automatically when participants had given a response or when a response deadline of 1,500 ms after stimulus presentation had expired.

Trials were grouped into runs of trials consisting of seven to nine trials each. Every trial run began with a switch trial in which target and distracter colors changed compared with the previous run. This switch trial was followed by a random number of six to eight consecutive repetition trials in which target and distracter colors remained constant (see Fig. 2B). Over the course of six blocks, participants completed 192 trial runs.

#### Design

The full design of Experiment 1 contained three experimental factors. The first factor, *repetition,* determined whether target and distracter colors in a given trial remained constant (repetition trials) or switched (switch trials) compared with the target and distracter colors in the previous trial. During switch trials, the color of target and distractor stimuli varied systematically as a function of the target color in the previous run. The nature of this dependence was varied by the second and third experimental factor. The second factor, *location of similarity*, varied which part of the current stimulus display depended on the previous target color: In the TS condition, the current *target* color was similar to the previous target color, whereas in the DS condition, the current *distracter* color was similar to the previous target color (see Fig. 2). The order of TS and DS switches was random with the constraint that four runs had to contain two TS and two DS switches to prevent biased sequences. The third factor, *color distance*, which was varied orthogonally to *location of similarity*, represented the distance between the previous target color and the current target color in the TS condition and the distance between the previous target color and the current distracter color in the DS condition. *Color distance* was varied in steps of 30° between 0° and 90° in color space (see Fig. 2B). Hence, in a 0° TS switch, the current target color was identical to the previous target color, while in a 0° DS switch, the current distracter color was identical to the previous target color. Conversely, in a 90° TS switch, the current target color was clearly different from the previous target color, while in a 90° DS switch, the current distracter color was clearly different from the previous target color (see Fig. 2B). The direction of the *color distance* manipulation in color space (e.g., from red to orange to yellow vs. from red to purple to blue; see Fig. 2A) was chosen at random.

To keep all other features of switch trials as comparable as possible, we took three additional measures. First, we applied three constraints to the selection of the color of the nonsimilar digit (i.e., the current distracter color in the TS condition and the current target color in the DS condition). In the TS condition, distracter colors differed (1) by at least 90° from the previous target color to prevent a persisting bias from previously task-relevant information (cf. Dreisbach & Goschke, 2004; Frisch et al., 2015; Longman et al., 2013); (2) by at least 30° from the previous distracter color to ensure that each switch in target colors also entailed a noticeable switch in distracter colors; and (3) by at least 90° from the current target color to make task-relevant and task-irrelevant information within each trial sufficiently distinct. Vice versa, in the DS condition, target colors differed (1) by at least 90° from the previous target color as DS and TS switches would overlap otherwise; (2) by at least 30° from the previous distracter color to prevent a persisting bias from previously task-irrelevant information (cf. Houghton & Tipper, 1994); and (3) by at least 90° from the current distracter color to make task-relevant and task-irrelevant information within each trial sufficiently distinct.

Second, we ensured that target and distracter digits indicated different responses (i.e., were response incompatible) for all trials preceding a switch. As incompatible responses are known to increase goal activation (Goschke, 2000), this was supposed to level out differences in goal shielding prior to a switch while at the same time maximizing the persisting aftereffects of previous goals during switch trials.

Third, all switch trials were kept response incompatible. This was necessary, as our paradigm measures how efficiently participants attend to a new target color while ignoring a distracter color. By linking these different sources of information to different responses, we ensured that conflict at the level of goal activation would be reflected in conflict during response selection and thus in performance.

In summary, Experiment 1 followed a 2 (repetition: repeat vs. switch of colors) × 2 (location of similarity: TS vs. DS condition) × 4 (color distance: 0° vs. 30° vs. 60° vs. 90° distance in color space) within-subject design, with response times (RTs) and errors as dependent variables. Additionally, the design was balanced regarding repetitions (vs. switches) of the location of the target stimulus (upper vs. lower) before vs. after the switch as well as regarding repetitions (vs. switches) of the to-be-given response (left vs. right) before versus after the switch.

#### Data preparation and statistical analyses

For ease of interpretation, speed and accuracy of performance were integrated into one measure by calculating the inverse efficiency score RT*^2^ (see Hughes et al., 2014; Townsend & Ashby, 1983; Vandierendonck, 2016) as the ratio of RT and accuracy for each participant in each combination of conditions. Results for RT and error rates can be found in the Supplement. Repetition trials mainly served to ensure that participants fully engaged with the current task goal, and performance during repetition trials was very high across all conditions (*M* = 549.87 ms/percent_correct) and contained no significant experimental effects (all *F* values < 4.2, all *p* values > 0.05). Therefore, the analyses reported here focus on switch trials only (but see the Supplement for the full analyses including repetition trials). Each subject completed 24 switch trials for each cell of the remaining 2 (location of similarity) × 4 (color distance) design matrix. Trials with missed deadlines (*M* = 0.75%, *SD* = 0.92%) or with RTs exceeding a *z*-score of 2 (*M* = 3.88%, *SD* = 1.69%) were excluded. Data were analyzed using MATLAB, R2010b (The MathWorks, Inc.) and IBM SPSS Statistics for Windows (Version 23; IBM Corp., 2015).

## Results

Based on the assumption that goal representations are continuous in representational space, we predicted a graded effect of color distance on performance, the direction of which should be mirror-inverted in the two location-of-similarity conditions.

**Fig. 3:**
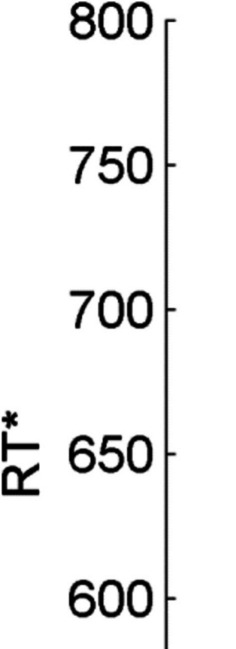
Results of Experiment 1 showing the inverse efficiency score RT* during switch trials as a function of *location of similarity* and *color distance.* Error bars represent standard errors

To test this hypothesis, we conducted a two-factorial repeated-measures analysis of variance (RM-ANOVA) including *location of similarity* and *color distance* as independent variables and RT* as a dependent variable. This analysis revealed a significant main effect for the factor *location of similarity*, *F*(1,24) = 74.24, *p* < 0.001, η_p_^2^ = 0.76, a nonsignificant main effect for the factor *color distance, F*(3,72) = 1.19, *p* = 0.32, η_p_^2^ = 0.05, as well as the predicted Location of Similarity × Color Distance interaction,* F*(3,72) = 15.79, *p* < 0.001, η_p_^2^ = 0.40. This interaction indicated that *color distance* affected performance, but that the nature of this effect depended on the *location of similarity*. Whereas in the TS condition decreasing the similarity between previous target and current target color had a negative effect on performance, decreasing the similarity between previous target and current distracter color in the DS condition had a positive effect on performance (see Fig. 3). To characterize the effect of distance in color space on performance further, we performed two additional analyses in which we tested the effect of *color distance* on performance in switch trials of the TS and DS conditions separately. Both analyses revealed significant effects of *color distance* on performance, *F*(3,72) = 8.18, *p* < 0.001, η_p_^2^ = 0.25 (for TS switch trials) and *F*(3,72) = 9.88, *p* < 0.001, η_p_^2^ = 0.29 (for DS switch trials). These effects were best described in terms of a linear trend, *F*(1,24) = 9.57, *p* < 0.01, η_p_^2^ = 0.29 (for TS switch trials) and *F*(1,24) = 17.74, *p* < 0.001, η_p_^2^ = 0.43 (for DS switch trials), or a quadratic trend, *F*(1,24) = 10.01, *p* < 0.01, η_p_^2^ = 0.29 (for TS switch trials) and *F*(1,24) = 7.57, *p* = 0.01, η_p_^2^ = 0.24 (for DS switch trials). However, neither of the two effects fit a cubic trend, *F*(1,24) = 0.40, *p* = 0.40, η_p_^2^ = 0.02 (for TS switch trials) and *F*(1,24) = 0.07, *p* = 0.80, η_p_^2^ = 0.00 (for DS switch trials). Taken together, Experiment 1 revealed a graded effect of *color distance* on performance in both *location of similarity* conditions, indicating that color goals are indeed represented in a continuous representational format. This effect was described best in terms of a monotonic trend.

## Discussion

In Experiment 1 we investigated the assumption that goal representations are continuous in representational space. Our results show that goal activation persists during goal switches: While participants’ performance profited considerably from complete goal repetitions (i.e., 0° switches in the TS condition), the need to abruptly ignore the previous target color (i.e., 0° switches in the DS condition) resulted in the worst performance across all cells of the design. More importantly, the size of both effects varied as a function of the distance between the previous color goal and the colors in the task display in a switch trial. While the performance benefit in the TS condition decreased as goal distance in color space increased, performance in the DS condition improved as the distance between previous target and current distracter color grew. The view that goal representations are discrete neural units that are independent from each other (e.g., Botvinick et al., 2001; Gilbert & Shallice, 2002; Shenhav et al., 2013; Verguts & Notebaert, 2008) does not predict such a graded effect. Instead, our results lend support to more recent approaches to cognition (e.g., Frisch et al., 2015; Johnson et al., 2008; Sandamirskaya et al., 2013; Wimmer et al., 2014) that assume information can be represented as distributed patterns of activation within continuous feature spaces in which they overlap to varying degrees. In addition, our results corroborate findings from the task- and set-shifting literature (Dreisbach & Goschke, 2004; Frisch et al., 2015; Goschke, 2000; Longman et al., 2013, 2014),

Beyond that, Experiment 1 also provides first indications precisely how goal activation spreads through this space. It is important to note that the relationship between distance in color space and performance in the two switching conditions of our set-shifting paradigm depends on the type of activation function goal representations follow as they spread through representational space. For instance, the prediction that increases in the distance in color space result in monotonically increasing or decreasing performance costs in the TS and DS conditions builds on the assumption that goal activation propagates through color space following a monotonic activation function. This is true for the bell-shaped activation function depicted in Fig. 1 that has its maximum at the to-be-coded feature value and then declines steadily toward zero with increasing distance. The assumption that goal representations may follow such a Gaussian distribution is closely related to findings on goal-directed attention (e.g., C. W. Eriksen & James, 1986; Hollingworth et al., 2012; Kravitz & Behrmann, 2008; LaBerge et al., 1997; Posner, 1980) showing that the acuity of the attentional spotlight is highest at the focus of attention and declines steadily the further away stimuli lie in the visual field. Likewise, the effects of the color distance manipulation in Experiment 1 were most pronounced at the 0° interval and declined steadily with increasing distance in color space. Thus, the pattern of results from Experiment 1 suggests that the spreading of goal activation through color space follows a simple, bell-shaped activation function.

**Fig. 4:**
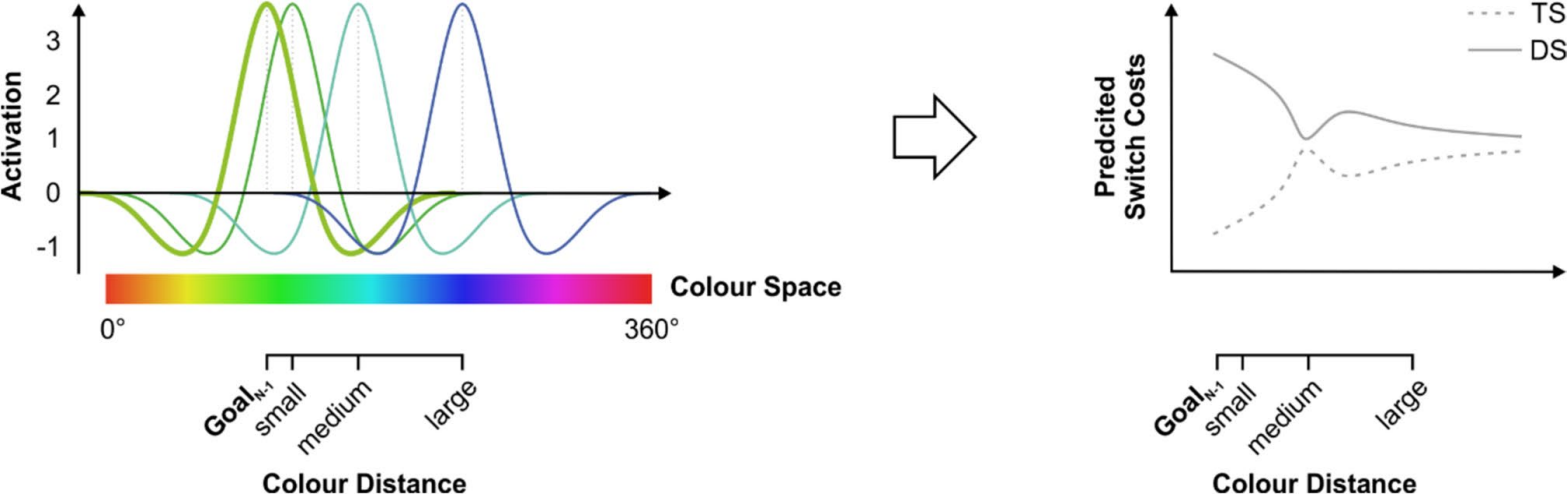
**Left:** Illustration of continuous color goal representations that follow a Mexican-hat-shaped activation function. **Right:** Resulting performance costs (e.g., response times, error rates) for goal switches of varying distance in color space in the target-similar (TS) and the distracter-similar (DS) conditions of the set-shifting paradigm. Under the assumption that goal representations follow a nonmonotonic (i.e., Mexican-hat-shaped) activation function, performance in both the TS and DS conditions should also show a nonmonotonic relationship with distance in color space. In particular, the overall decline in switching performance with increasing color distance in the TS condition should be interrupted by a transitory dip when the current target color lies within the inhibitory “brim” of the previous color goal. Vice versa, the overall improvement in switching performance in the DS condition should exhibit a transitory performance peak when the current distracter color lies in the inhibitory “brim” of the previous color goal. (Color figure online)

While such a simple pattern of activation distribution appears plausible, the spreading of goal activation through representational space may be more complex. In the realm of selective attention, it has been argued that an attentional gradient that decreases monotonically with increasing distance to the current focus of attention may not suffice to reliably differentiate task-relevant from task-irrelevant information (see, e.g., N. G. Müller et al., 2005; Tsotsos et al., 1995). As stimuli lying close to the current focus of attention receive only slightly less activation than stimuli lying within the current focus of attention, a monotonically decreasing attentional gradient bears the risk of confusing task-relevant with task-irrelevant but similar stimuli. Accordingly, it has been proposed that attentional selection may incorporate inhibitory processes (Tsotsos et al., 1995, 2001) in addition to the purely excitatory, bell-shaped peak proposed by earlier accounts (e.g., Posner, 1980). This center-surround inhibition (also called a Mexican-hat shape; see Fig. 4) is thought to reduce interference by suppressing confusable stimuli that are similar but not identical to the current focus of attention. Indeed, a number of recent studies have observed such center-surround inhibition for both spatial (e.g., Hopf et al., 2006; Mounts, 2000; N. G. Müller et al., 2005, p. 200; Pinsk et al., 2004; Slotnick et al., 2003) and feature-based attention (e.g., Störmer & Alvarez, 2014). More importantly, a recent study by Kiyonaga and Egner (2016) suggests that these activation dynamics might bear relevance for the representation of goals as well. In particular, the authors tested whether the inhibitory center-surround mechanisms observed in location and feature-based attention were involved in the maintenance of information in working memory, too. To this end, they measured how much holding a color in working memory interferes with the processing of an unrelated task in which distracter stimuli appeared in colors similar to the memorized item. In line with a Mexican-hat-shaped distribution of activation in working memory, the authors found a nonmonotonic, tripartite (i.e., cubic) effect: While interference was strongest when distracters matched the item in working memory (i.e., lay within the excitatory top of the hat), interference was drastically reduced when distracters were relatively similar but not identical to the memory item (i.e., lay win the inhibitory brim of the hat) only to increase again for distracters that were even further away, outside of the inhibitory brim.

With regard to the question which activation function—bell-shaped or Mexican-hat shaped—underlies goal representation, the monotonic relationship between distance in color space and performance that we observed in both conditions of our set-shifting paradigm points toward the former. A Mexican-hat-shaped pattern of central activation and peripheral inhibition should have produced behavioral patterns similar to the ones reported by Kiyonaga and Egner (2016): If center-surround inhibition played a role in the representation of color goals, this should have resulted in nonmonotonically increasing (i.e., cubic or quartic) relationships between distance and performance in the TS condition, and a nonmonotonically decreasing (i.e., cubic or quartic) relationships between distance and performance in the DS condition. This is because colors in the close vicinity of the previous goal representation would receive an activation benefit, while colors lying in the “brim” of the activation function would be suppressed below baseline and would, thus, suffer from a competitive disadvantage. As this inhibitory effect levels off when the distance between a color and the previous goal is increased further, a center-surround mechanism of goal activation would cause a renewed rise in performance, resulting in the wave-like pattern that is depicted in Fig. 4.

While we know that a Mexican-hat-shaped activation function should result in a transitory dip (in the TS condition) or peak (in the DS condition) in performance, neither the depth of this brim (i.e., the size of the inhibitory effect surrounding a peak) nor its precise location (i.e., whether it lies 5° or 50° degrees away from the current target color in feature space) are known. On this account, Experiment 1 has three important limitations that need to be addressed if we aim to distinguish between the two activation functions. First, while our sample of 25 participants is well in line with the standards of behavioral experiments in cognitive psychology and considerably larger than in other studies addressing similar research questions (Kiyonaga & Egner, 2016; Störmer & Alvarez, 2014), it is possible that it is still too small to detect the potentially modest effects of center-surround inhibition on overt behavior. A second factor that may have resulted in an overall too low level of experimental power is the arguably low level of sensitivity of our main dependent measures. While previous studies have demonstrated center-surround inhibition in standard markers of performance such as RTs and error rates in pure working memory tasks, the effects produced in our set-shifting design may be less pronounced such that they remain undetected in such coarse measures of behavior. The third and final limitation pertains to the unknown location of the effects of interest. As we do not know at which distance precisely the suspected inhibitory brim of a Mexican hat unfolds, it is possible that the levels of the level of similarity manipulation used in experiment 1 (30°, 60°, and 90°) may have been too coarsely chosen to detect the resulting effects. To address each of these three limitations, we conducted Experiment 2 in which we replicated study one within a larger sample and relying on both a more fine-grained manipulation of goal similarity and a more sensitive dependent measure.

## Experiment 2

The aims of Experiment 2 were (1) to provide further evidence for the continuity of goal representations in representational space in a design similar to Experiment 1 and (2) to adjust factors of this design that may have prevented us from observing effects of Mexican-hat-like, center-surround inhibition in the results of Experiment 1. Accordingly, we upped our sample size to increase the power to detect small effect. Based on the observed effect size for the interaction of Location of Similarity × Color Distance in Experiment 1 of η_p_^2^ = 0.40, an a priori power analysis with a power of 0.80 revealed a target sample size of *N* = 30 (Faul et al., 2007). Moreover, in an attempt to increase the sensitivity of our main dependent measure in addition to overall statistical power, the response mode of Experiment 2 was changed. Instead of indicating their answer by pressing left and right buttons on a keyboard, participants responded by moving a computer mouse into the left and right corners of the screen (see McKinstry et al., 2008; Scherbaum et al., 2010, 2015; Spivey et al., 2005). Such mouse-tracking methods have been successfully used to investigate cognitive processing in task switching paradigms (e.g., Huette & McMurray, 2010; Ye & Damian, 2022). Finally, Experiment 2 increased the resolution of the level of similarity manipulation. Instead of varying intergoal distance in steps of 30° degrees in color space, steps of 20° were applied in Experiment 2 to allow for a more fine-grained examination of the spreading of goal activation through representational space.

### Methods

#### Participants

Thirty participants (22 women, *M*_Age_ = 23.77 years) from Technische Universität Dresden took part in the experiment and received 7€ or course credits for their participation. All participants had normal or corrected-to-normal vision and color vision and had not taken part in Experiment 1. The study was approved by the ethics committee of the Technische Universität Dresden and all participants gave informed consent in accordance with the Declaration of Helsinki.

#### Apparatus and stimuli

The setup and stimuli used in Experiment 2 were identical to the ones reported for Experiment 1, except for three changes. First, instead of selecting hues for cues, target, and distracter stimuli in steps of 30° relative to a random starting point in HSV color space, hues were selected in steps of 20° in Experiment 2. Second, instead of pressing left and right buttons on a keyboard, participants gave their responses by moving a computer mouse (Logitech Laser Mouse) into the upper left or right corners of the screen. Third, for responses to be given via movements across the screen, one start and two response boxes—all white—were added to the setup. The start box was a horizontal box (60 pixels high) that was located at the bottom of the screen and extended across its entire width (i.e., 1,280 pixels). The response boxes were rectangles of 224 × 100 pixels located in the upper left and right corners of the screen.

#### Procedure

While the overall procedure of Experiment 2 was similar to Experiment 1, some adjustments were necessary to use the mouse as response device (see Frisch et al., 2015; Scherbaum et al., 2010, 2015; Spivey et al., 2005). Each trial consisted of three phases. First, to standardize the starting point of each movement, participants had to move the mouse into the starting box at the bottom of the screen within a deadline of 1.5 s after their previous response to make the fixation cross appear. Second, participants had to start moving their mouse upwards within a deadline of 1 s. As soon as they had passed a virtual line 60 pixels above their starting point, the fixation cross was replaced by the colored, rectangular frame cueing the current target color. This frame remained on-screen for 200 ms until it, third, was replaced by the target and distracter digits appearing one above the other in the center of the screen. Subjects were instructed to respond as quickly and accurately as possible to the magnitude of the digit presented in the target color by moving the mouse into the upper left (< 5) or right (> 5) corner of the screen within a deadline of 1.5 s. Response times reflected the time elapsed between stimulus presentation and reaching a response box. The next trial was initiated as soon as participants touched the start bar at the bottom of the screen again or when participants missed one of the three deadlines.

### Design

While *location of similarity* was varied like in Experiment 1, the resolution of the *color distance* manipulation was increased to include four levels representing steps of 20° between 0° and 60° in color space. Thus, Experiment 2 followed a 2 (location of similarity: TS vs. DS) × 4 (color distance: 0° vs. 20° vs. 40° vs. 60°) within-subject design. Over the course of five blocks, each subject completed 160 switch trials with 20 trials for each cell of this design matrix. Again, the design was also balanced regarding repetitions (vs. switches) of the location of the target stimulus (upper vs. lower) before vs. after the switch as well as regarding repetitions (vs. switches) of the to-be-given response (left vs. right) before versus after the switch.

### Data preparation

Following the same rationale as discussed for Experiment 1, analyses focused on behavior in switch trials (but see the Supplement for the full analyses including repetition trials). Trials with missed deadlines (*M* = 1.08%, *SD* = 1.14%) or with RTs exceeding a *z*-score of 2 (*M* = 1.13%, *SD* = 1.43%) were excluded. Static measures of performance (i.e., RT and accuracy) were combined into the inverse efficiency score RT* (see Townsend & Ashby, 1983; Vandierendonck, 2016; see Supplement for results on RT and error rates). To assess the level of attraction toward the distracter stimulus during switch trials, we calculated the area under the curve (AUC) for each movement trajectory (see, e.g., Scherbaum et al., 2010; Spivey et al., 2005). To this end, mouse movements of correct responses were aligned to a common starting position and mirrored such that positive values always reflected correct responses. AUCs were then calculated as the area between the actual trajectory and an ideal trajectory (a straight line between the start and finish point of the trajectory). Because trajectories can only be analyzed meaningfully for correct responses, the proportion of our experimental effects that is reflected in erroneous responses is left unaccounted for. To factor in both measures of performance, we calculated AUC*, a composite score like RT* that results from dividing the mean AUC by the mean accuracy in each cell of the design. Results for AUC can be found in the supplement. Data were analyzed using MATLAB, R2010b (The MathWorks, Inc.) and IBM SPSS Statistics for Windows (Version 23; IBM Corp.).

## Results

### RT*

**Fig. 5:**
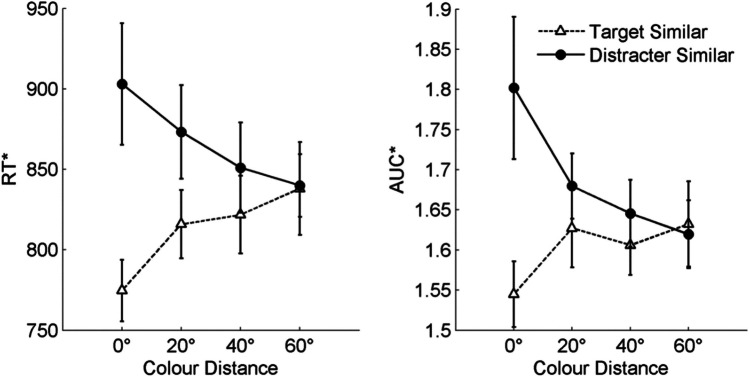
Results of Experiment 2 showing the inverse efficiency score RT* (*left*) and AUC* (*right*) for switch trials as a function of *location of similarity* and *color distance*. Error bars represent standard errors

The pattern of results observed for RT* in Experiment 2 was similar to the one observed in Experiment 1 (see Fig. 5). A two-factorial RM-ANOVA revealed a significant main effect for the factor *location of similarity*, *F*(1,29) = 15.76, *p* < 0.001, η_p_^2^ = 0.35, a nonsignificant main effect for the factor *color distance, F*(3,87) = 0.16, *p* > 0.90, η_p_^2^ = 0.01, as well as the predicted Location of Similarity × Color Distance interaction*, F*(3,87) = 5.85, *p* = 0.001, η_p_^2^ = 0.17. Again, the latter effect indicated that the distance in representational space between a former goal and currently task-relevant (TS) or task-irrelevant (DS) information affects how easily this information can be attended or ignored, respectively. To characterize the nature of this effect further, two RM-ANOVAs were performed on switch trials in the TS and DS conditions, separately. For switch trials in the TS condition, this analysis revealed a significant main effect of *color distance, F*(3,87) = 7.48, *p* < 0.001, η_p_^2^ = 0.21. Post hoc contrasts showed that performance deteriorated linearly with increasing distance between previous and current color goal in color space, *F*(1,29) = 13.37, *p* = 0.001, η_p_^2^ = 0.32, and that the basis of this relationship could not be better described by a quadratic or cubic term (all *p* values > 0.18, all *F* values < 1.91). For switch trials in the DS condition, the expected effect of *color distance* on performance missed statistical significance, *F*(3,87) = 2.52, *p* = 0.06, η_p_^2^ = 0.08. Nevertheless, post hoc contrast showed that the relationship between *color distance* and performance could be described in terms of a linear, *F*(1,29) = 4.95, *p* = 0.034, η_p_^2^ = 0.15, but not quadratic or cubic trend (all *p* values > 0.64, all *F* values < 0.22), suggesting that the distracting effect of task-irrelevant information decreased linearly the more the current distracter color differed from the previous target color in terms of their distance in color space.

### AUC*

The pattern of results observed for the movement-based measure of performance closely followed those observed for RT*. An RM-ANOVA showed a significant main effect of *location of similarity*, *F*(1,29) = 18.30, *p* < 0.001, η_p_^2^ = 0.39, a nonsignificant main effect for the factor *color distance, F*(3,87) = 0.87, *p* > 0.45, η_p_^2^ = 0.03, as well as the predicted Location of Similarity × Color Distance interaction*, F*(3,87) = 3.70, *p* = 0.02, η_p_^2^ = 0.11. Follow-up analyses on switch trials in the two *location of similarity* conditions separately revealed that the effect of *color distance* on AUC* was best described by linear trends in both conditions, *F*(1,29) = 5.77, *p* = 0.02, η_p_^2^ = 0.17 in the TS condition and *F*(1,29) = 4.61, *p* = 0.04, η_p_^2^ = 0.14 in the DS condition, as higher-order trends did not reach significance (all *p* values > 0.17, all *F* values < 2.00).

## Discussion

In Experiment 2, results obtained from both static (i.e., RT*) and dynamic (i.e., AUC*) measures of performance overlapped closely and replicated the core findings from Experiment 1. We found (1) evidence for persisting goal activation during goal switches (e.g., Dreisbach & Goschke, 2004; Frisch et al., 2015; Goschke, 2000; Longman et al., 2013, 2014) that (2) varied systematically as a function of goal-to-goal overlap in color space, a relationship that was (3) best described in terms of a monotonic (i.e., linear) trend and did not fit a nonmonotonic relationship. Taken together, the findings from Experiment 2 further highlight the continuous impact of goal representations on performance, thus providing an important extension of standard models of cognitive control that implemented goals as discrete entities (e.g., Botvinick et al., 2001; Gilbert & Shallice, 2002; Shenhav et al., 2013; Verguts & Notebaert, 2008). Moreover, the fact that the association between color distance and performance in the two switching conditions was again best described in terms of a monotonic, linear trend is more consistent with the idea that goal activity follows a bell-shaped than a Mexican-hat-shaped activation function. This is a particularly important finding because Experiment 2 has been designed to specifically address several issues that may have prevented us from observing center-surround inhibition in Experiment 1 by adding subjects to our sample, applying a more fine-grained gradation variation of distances in color space, and including mouse-tracking as a potentially more sensitive dependent measure.

Nevertheless, excluding an explanation based on the absence of effects in observed behavior is, of course, difficult. This is particularly true bearing in mind that the objection regarding the level of resolution of the level of similarity raised for Experiment 1 still applies to Experiment 2. Even though distances in color space were now manipulated in steps of 20° instead of 30°, it cannot be excluded that the inhibitory effects of interest unfold on an even smaller level. Therefore, we conducted a third experiment in which the resolution of goal-to-goal distance in representational space was increased once again to ensure that nonmonotonic effects could manifest. Moreover, in an effort to increase experimental power and the usage of more sensitive dependent measures of performance, Experiment 3 took a psychophysical approach by investigating a small number of subjects with a very high number of trials.

## Experiment 3

Experiment 3 followed the same rationale and objectives as Experiments 1 and 2, with three important changes. First, Experiment 3 further increased the resolution of the *color distance* manipulation such that seven intergoal distances ranging between 0° and 60° in steps of 10° in color space were covered. Second, as the change in answering devices in Experiment 2 led to similar results, we returned to the setup of Experiment 1 by having participants respond via button presses. Third, and most importantly, Experiment 3 aimed to increase the validity of the acquired data by following an approach inspired by psychophysical methods. Instead of having a large sample of participants provide a small amount of data, Experiment 3 relied on a smaller number of participants who each provided a large number of trials. To prevent effects of fatigue, the experiment was conducted across four sessions of one hour each, with a maximum of 2 days in-between sessions. Accordingly, a more accurate estimation of the statistical variance within the cells can be achieved.^3^ The larger number of trials per cell and subject has the advantage that more random noise is averaged out and, thus, that the resulting mean will represent the “true effect” of the underlying (combination of) factors more accurately. Hence, upping the number of trials per cell should increase the overall sensitivity of our experimental design.

### Methods

#### Participants

Fifteen participants (11 women, *M*_Age_ = 20.33 years) from Technische Universität Dresden took part in the experiment and received 40€ or course credits for their participation. All participants had normal or corrected-to-normal vision and color vision and had not taken part in Experiments 1 or 2. The study was approved by the ethics committee of the Technische Universität Dresden and all participants gave informed consent in accordance with to the Declaration of Helsinki.

#### Apparatus and stimuli

The setup and stimuli used in experiment 3 were identical to the ones used in Experiment 1, with one exception: Instead of selecting hues for cues, target, and distracter stimuli in steps of 30° (and 20° in Experiment 2) relative to a randomly chosen starting point in HSV color space, colors were selected in steps of 10° in Experiment 3.

### Procedure

Each trial followed the same procedure as in Experiment 1: Participants saw a fixation cross for 500 ms, 700 ms, or 900 ms followed by a rectangular frame indicating the target color for the current trial. After 200 ms, this cue was replaced by the target and distracter digits appearing in different colors that remained on-screen until participants gave a response by pressing a left versus right button on a standard keyboard or a response deadline of 1,500 ms ran out.

Each participant completed four, hour-long testing sessions on different days, with a minimum of 24 and a maximum of 72 h between consecutive sessions. In each session, participants completed four blocks of trials with self-paced breaks in-between. Each block consisted of 56 runs of trials with four runs of each block representing one of the 14 combinations of the factors *location of similarity* and *color distance*. Across all four sessions of the experiment, each participant completed a total of 896 runs of trials, with each run consisting of one switch trial and a random number of six to eight repetition trials. All other aspects of the procedure were identical to Experiment 1.

### Design

Experiment 3 followed a 2 (location of similarity: TS vs. DS) × 7 (color distance: 0° vs. 10° vs. 20° vs. 30° vs. 40° vs. 50° vs. 60°) within-subjects design. Each subject completed 896 switch trials, with 64 switch trials for each cell of the design matrix. Additionally, the design was balanced regarding repetitions (vs. switches) of the location of the target stimulus before versus after the switch as well as regarding repetitions (vs. switches) of the to-be-given response before versus after the switch. Data were analyzed using Matlab, R2010b (The MathWorks, Inc.) and IBM SPSS Statistics for Windows (Version 23; IBM Corp.).

### Data preparation

Again, analyses focused on RT* (Townsend & Ashby, 1983) in switch trials (but see the Supplement for the full analyses including repetition trials as well as results for RT and accuracy). Trials with missed deadlines (*M* = 0.26%, *SD* = 0.29%) or with RTs exceeding a *z*-score of 2 (*M* = 3.82%, *SD* = 0.70%) were excluded.

## Results

**Fig. 6:**
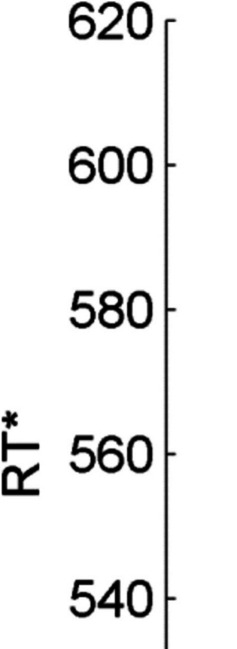
Results of Experiment 3 showing the inverse efficiency score RT* for switch trials as a function of *location of similarity* and *color distance*. Error bars represent standard errors

As in Experiments 1 and 2, a, RM-ANOVA revealed a significant main effect for the factor *location of similarity*, *F*(1,14) = 8.96, *p* = 0.01, η_p_^2^ = 0.39, a nonsignificant main effect for the factor *color distance, F*(6,−84) = 0.67, *p* = 0.68, η_p_^2^ = 0.05, as well as the predicted Location of Similarity × Color Distance interaction*, F*(6,84) = 3.59, *p* = 0.003, η_p_^2^ = 0.20 (see Fig. 6). Following up on this effect with separate RM-ANOVAs for switch trials in the TS and DS conditions, separately, we found that, as predicted, performance in the TS condition worsened the more dissimilar successive color goals were, *F*(6,84) = 4.30, *p* < 0.001, η_p_^2^ = 0.24. This relationship was best described by a linear, *F*(1,14) = 20.81, *p* < 0.001, η_p_^2^ = 0.60, or quadratic trend, *F*(1,14) = 8.35, *p* = 0.012, η_p_^2^ = 0.37, and did not fit any higher-order trend (all *p* values > 0.20, all *F *values < 1.74). While performance in switch trials of the DS condition descriptively appeared to follow a similar yet inverse pattern with performance improving with increasing color distance, this effect failed to reach significance, *F*(6,84) = 0.92, *p* = 0.49, η_p_^2^ = 0.06. Accordingly, a linear trend fitted to the data missed significance, *F*(1,14) = 3.94, *p* = 0.067, η_p_^2^ = 0.22, and trends of higher order were far from statistical reliability (all *p* values > 0.31, all *F* values < 1.10). When performing these analyses separately for the first two and the last two sessions, the effect of color distance was less pronounced and no longer significant in the last two sessions, indicating potential fatigue or floor effects (see the Supplement for details).

## Discussion

Supporting the results from Experiments 1 and 2, Experiment 3 provided further evidence that goal representations are continuous in representational space. Taking an approach inspired by psychophysics that aimed at reducing the amount of error variance in the data by collecting a large number of trials from a small number of participants and increasing the resolution of the *color distance* manipulation one more time, the overall pattern of results in Experiment 3 was largely consistent with the findings reported earlier. As expected, *color distance* covaried systematically with performance in the two switching conditions. Moreover, the linear relationships between goal-to-goal distance and performance in color space observed in our data is more consistent with bell-shaped (i.e., monotonic) spreading of goal activation in representational space than with a Mexican-hat-shaped (i.e., nonmonotonic) one. This is at odds with findings from the working memory literature suggesting that inhibitory processes take part in the selection of internal representations in color working memory (Kiyonaga & Egner, 2016; Störmer & Alvarez, 2014). It must be noted, however, that the effects of interest were overall more pronounced in the TS than in the DS conditions, for which only the linear trend but not the main effect of *color distance* reached statistical reliability. In addition, the effect of color distance decreased over the course of the experiment and was no longer significant during the last two sessions. It thus appears that the psychophysical approach taken in Experiment 3 has weakened the effect of previous goal representations on ongoing behavior. Particularly, participants of Experiment 3 who worked extensively on our paradigm for 4 h in total seem to be better at ignoring previously task-relevant information during goal switching than participants in Experiments 1 and 2 who only completed a single hour of the task. While this might indicate additional strategic or learning-based effects on goal switching after heavy practice, the clear linear effects of *color distance* on performance in the TS condition show that behavior is still affected by the distance of consecutive goal representations in color space.

## General discussion

The purpose of our investigation was to explore how goals are represented, a question that is often neglected in research on motivation and volition. We explored the idea of more dynamic approaches to cognition that goals can also be described as patterns of neural activation which overlap and interact to varying degrees within continuous mental spaces (e.g., Dshemuchadse et al., 2015; Frisch et al., 2015; Johnson et al., 2009a, 2009b; Lins & Schöner, 2014; Sandamirskaya et al., 2013; Wimmer et al., 2014). To test this assumption, we conducted three experiments with a novel set-shifting task in which participants had to switch repeatedly between different color goals that varied systematically in their distance in color space according to two switching conditions. In the target switch condition, new target colors were more or less similar to the former color goal. In the distracter switch condition, new distracter colors were more or less similar to the former color goal. We hypothesized that if goal representations were continuous in representational space, performance should vary continuously as a function of goal-to-goal and goal-to-distracter overlap in both switching conditions. However, if goal representations were discrete, we would expect a binary effect of color distance on performance with complete color repetitions having a particularly large impact on behavior and all other changes in target or distracter colors having similar effects on performance, irrespective of their distance in color space.

The results of three experiments showed that performance varied continuously as a function of color distance between both task-relevant (in the target switch condition) and task-irrelevant (in the distracter switch condition) information and previous color goals. This pattern of results extends the image of goal representations in standard accounts of cognitive control (Botvinick et al., 2001; Gilbert & Shallice, 2002; Shenhav et al., 2013; Verguts & Notebaert, 2008), which model goal representations as sparsely connected, single neural units. It is more compatible with continuous approaches to cognition (e.g., Dshemuchadse et al., 2015; Frisch et al., 2015; Gärdenfors, 2004; Johnson et al., 2009a, 2009b; Lins & Schöner, 2014; Sandamirskaya et al., 2013; Wei et al., 2012; Wimmer et al., 2014), which hold that information in the cognitive system is represented by diffuse peaks or bumps of activation that spread through continuous representational spaces created by the populations of neurons coding specific stimulus features like location, tilt, or color. From this perspective, the decrease in performance observed with increasing color distance in the target switch condition follows naturally because the portion of shared activation between the two peaks representing the previous and current goal representation in a switch trial grew smaller. This increases the time needed for putting the current goal representation in a state of activation. Conversely, the increase in performance observed in the distracter switch condition with increasing color distance occurred because the portion of shared activation between the peaks representing the previous task goal and currently interfering stimuli was reduced, thus diminishing these color’s persisting distracting effect on behavior.

These results resonate well with recent findings from the working memory literature which point toward a distributed format of representation in this part of the cognitive system (e.g., Guérard & Tremblay, 2011; Johnson & Spencer, 2016; Kiyonaga & Egner, 2016; Rademaker et al., 2015; Störmer & Alvarez, 2014; Van der Stigchel et al., 2007). While goals are generally considered to be held in working memory as well (e.g., Goschke, 2000; Gruber & Goschke, 2004; Miller & Cohen, 2001), our data adds to these findings in two important ways. First, while the existing body of work primarily focused on the effects of distance on representations that are maintained in working memory for later retrieval (e.g.,Johnson et al., 2008, 2009a, 2009b; Kiyonaga & Egner, 2016; Spencer & Hund, 2003), our studies asked whether the same principles apply to goals, that is, to working memory representations that are engaged in the current task to guide perception and action toward the completion of a task (e.g., Gruber & Goschke, 2004; Miller & Cohen, 2001). Our findings clearly show that the efficiency of this guidance hinges on the amount of representational overlap between a task goal and both task-relevant and task-irrelevant features of the environment. Thus, our work demonstrates that representational continuity not only resides within memory but also shapes the way we interact with the outside world.

A second important way in which our work differs from existing studies concerns the relevance of the representations under study for the ongoing task. Previous research has focused on the question whether and how distance in representational space affects representations that are held in working memory as task-relevant information. To this end, they had participants memorize multiple stimulus features like different locations, tilts, or colors and showed that the values reported upon recall varied systematically as a function of their distance in representational space. This indicates that memory representations that were close in representational space had been draw toward each other or even merged during active maintenance (Johnson et al., 2008; Johnson et al., 2009a, 2009b; e.g., Johnson & Spencer, 2016; Kiyonaga & Egner, 2016; Rademaker et al., 2015; Spencer & Hund, 2003; Van der Stigchel et al., 2007; but note that this can also result in repulsion effects, see Bae & Luck, 2017; Johnson et al., 2022; Scotti et al., 2021). Going further, we explored whether representational continuity also applies for task representations that are no longer task-relevant. By showing that this is indeed the case, our work nicely complements studies from the task-switching literature that explore the persisting effects of previous task representations on current performance (see Kiesel et al., 2010; Koch et al., 2010, for an overview), a phenomenon that is often referred to as task-set inertia (e.g., Allport & Wylie, 2000). Our study adds to this body of research by showing that the extent of task inertia depends on the level of similarity between the decaying goal representation and current features of the environment. Within the dynamic approach to cognition, our study thus adds evidence that goal representation are continuous in both representational space and time (see Frisch et al., 2015; Scherbaum et al., 2008; Spivey & Dale, 2004).

A question that directly follows from the observation that goals are continuous in representational space is how precisely goal activation spreads through this space. Our design allows us to differentiate two potential answers to this question. On the one hand, this could be a bell-shaped activity distribution that peaks at the current target value and decreases continuously to zero with increasing target distance, which can be derived from classic accounts of attention (e.g., C. W. Eriksen & James, 1986; Hollingworth et al., 2012; Kravitz & Behrmann, 2008; LaBerge et al., 1997; Posner, 1980). On the other hand, this could be a center-surround, Mexican-hat-shaped activation function. The Mexican-hat-shaped activation function is characterized by an additional inhibitory component that suppresses feature values close to but not identical to the current target. This is supported by modelling efforts (e.g., Tsotsos et al., 1995, 2001) and findings on the dynamics of goal-directed attention and working memory (e.g., Hopf et al., 2006; Kiyonaga & Egner, 2016; Mounts, 2000; N. G. Müller et al., 2005; Störmer & Alvarez, 2014). These two views make different predictions regarding the effects of color distance on performance in our design: While a monotonic, bell-shaped activation function should result in a monotonic association between color distance and performance, a nonmonotonic, Mexican-hat-shaped activity distribution should also produce a nonmonotonic relationship between color distance and performance reflecting the inhibitory “brim” it contains.

Our study shows clear support for a bell-shaped activation function. In both switching conditions across all three experiments, distance in representational space had linear or quadratic effects on performance. In no experiment or condition did we observe indications of a transitory dip or peak in performance that would be indicative of a center-surround inhibitory mechanism being at work in our paradigm. Thus, our data support the idea of a bell-shaped activation function (e.g., Posner, 1980) and yield no indication of a Mexican-hat-shaped distribution of activation within representational space. In this point, our results contradict recent evidence that has shown center-surround inhibition for color representations in working memory (Kiyonaga & Egner, 2016; Störmer & Alvarez, 2014).

There are several potential explanations as to why our results did not yield similar effects indicative of center-surround inhibition. First, the level of resolution of the *color distance* manipulation in our studies may have been too coarse to reflect inhibitory processes operating in the cognitive system. As is explained in greater detail above, the precise distance in representational space at which a potential inhibitory mechanism unfolds can only be speculated on. We tried to counter this uncertainty by gradually reducing the distance between consecutive levels of our color manipulation from 30° in Experiment 1 to 10° in Experiment 3, the distance in representational space at which center-surround inhibition has been observed in color working memory before (Kiyonaga & Egner, 2016). Still, this may not have covered the precise range of values at which center-surround inhibition occurs in our task. Second, although effect sizes and power are within the expected range, the absence of inhibitory effects in our experiments may reflect a lack of experimental power. As the effects of center-surround inhibition are presumably small in size, we upped our sample size and employed mouse movements as a potentially more sensitive response mode in Experiment 2. In Experiment 3, we applied an approach that was inspired by psychophysics which aimed to minimize both within- and between-person error variance by collecting a large number of trials from a selected number of subjects that led to a 33% stronger statistical power. Nevertheless, our data only reliably show the linear and quadratic effects that would be expected from a bell-shaped spreading of goal activation through representational space. Third, it is possible that our results emerge due to the way that longer-lasting memory traces of the previous target color impact current working memory. Long-term effects could have a more graded bell-shape curve (Lipinski et al., 2010); perhaps this affects how previous target colors are represented in active working memory. Fourth, one could speculate that inhibition serving to stabilize a certain activation does not necessarily have to be Mexican-hat shaped but could also be implemented by a form of global inhibition which, again, follows a bell-shaped curve that outside of the activation part reflects inhibition along most parts of the metric dimension. While the Mexican-hat shape could allow for local inhibition and hence for multiple distantly active goals, such a global inhibition would only allow for very few, if not only one active goal.

Still, our study does not rule out that inhibition has a share in the maintenance and shielding of goals. As has been noted above, one core feature of the work presented here is that it explores the nature of goal representations by examining the aftereffects of goals that are no longer task relevant. While a considerable amount of research has shown that goal representations continue to affect behavior after they have been abandoned (e.g., Allport & Wylie, 2000; Dreisbach & Goschke, 2004; Frisch et al., 2015; Longman et al., 2013), very little is known about the precise fate of outdated representations. For instance, it is perfectly conceivable that the excitatory and inhibitory sub-components that make up a Mexican-hat-shaped activation function are both at work during the active maintenance of a goal representation but that they decay at different time-scales once this goal is no longer relevant. Accordingly, it is possible that the two studies that consistently found center-surround inhibition for feature-based working memory and goal-directed attention (Kiyonaga & Egner, 2016; Störmer & Alvarez, 2014) did so because inhibition is only at work while a representation is actively held in working memory but quickly disappears afterwards. In contrast, the excitatory component of the representation is generally assumed to be much larger and may therefore persist a lot longer in the system, driving behavior even after new tasks have become relevant. Our findings only provide initial evidence regarding this intriguing idea, showing no evidence for persisting center-surround inhibition in our design. Future research is needed to understand which excitatory and inhibitory subprocesses are involved in the representation of goals and how these processes evolve throughout goal setting, maintenance, and decay (see Frisch et al., 2015). As possible solutions to tackle this question we propose to reduce the number of repeat trials in order to enhance the number of switch trials (enhancing statistical power) or by examining the effect in task paradigms other than the classical set-shifting task (e.g., the prime-probe task).

While our results remain ambiguous regarding the question about how precisely goal activation spreads through color space, they clearly demonstrate that color goals can be represented continuously. An important issue for future research will be to explore whether representational continuity is a general feature of goal representations or not. This important question will need to be addressed on at least two levels. First, on a basic level, it remains to be shown whether the continuity principle also holds for other goal-relevant stimulus features apart from color. Ultimately, the dynamic approaches underlying this work predict that all sensory and motor information in the cognitive system is represented in a continuous fashion (see, e.g., Frisch et al., 2015; Johnson et al., 2009a, 2009b; Lins & Schöner, 2014; Sandamirskaya et al., 2013). Accordingly, future studies will have to examine whether and to what extent representational continuity also applies to goals that require focusing on other low-level stimulus features such as tilt, location, or size, as well as on nonvisual features such as pitch and volume, pressure, or even taste and smell.

Second, on a broader level, it remains to be shown whether the continuity principle also applies to more complex types of goal representations. The work presented here focused on a conception of goals as higher-order representations that guide cognitive processing toward goal-relevant and away from distracting information (see, e.g., Miller, 2000). While this is a common perspective on goals in cognitive control research, other fields in psychology use the goal construct in different, broader terms (cf. Austin & Vancouver, 1996; Elliot & Fryer, 2008). For example, in the social psychological literature of motivation and volition (see, e.g., Kruglanski & Köpetz, 2009, for an overview), goals are often conceptualized as representations of desired end states (e.g., to water the plant on my desk) that are closely linked to the means to achieve them (e.g., to use the watering can from my kitchen). It is an intriguing question whether such complex goal representations are represented continuously as well. On a phenomenological level, such continuity should manifest in behavioral effects alike the ones observed in our studies. Indeed, we all experience situations where goals (e.g., the goal to water the plant on my desk) become unstable (e.g., because I must leave the desk to get the watering can from the kitchen) and are either forgotten (e.g., arriving in the kitchen and asking myself why I came here in the first place) or overridden by alternatives. A continuous perspective on goals would predict that such a “hijacking” becomes more likely with increasing representational overlap between the original goal and the overriding response alternative (e.g., the goal to water my plants should be more likely to be overridden by an impulse to drink the glass of water that sits on the table than by an impulse to eat a sandwich).

Notwithstanding these theoretical contributions to the scientific field of cognitive control processes, it is important to note that goals are not necessarily always represented in a continuous manner. Even though several model approaches that already implemented goals as continuous representations (Buss et al., 2013; Spencer et al., 2001; Thelen et al., 2001), it is open for future examinations when and how cognitive processes emerge in either discrete or continuous ways, which might also depend on the situational circumstances and tasks. Indeed, while we argue that behavioral and modelling evidence points toward a continuous representation of goals, our current study only probed behavioral performance. It is therefore possible that the continuity effects we found on performance emerged due to continuity on a low-dimensional feature level and not due to continuity on a high-dimensional goal level. To directly probe this goal level, it is necessary to investigate brain states directly. There is a growing body of research (see Rissman & Wagner, 2012; Xue, 2018, for an overview) demonstrating representational continuity for episodic memory, the memory subsystem that is most likely to be involved in the representation of goals as desired end states. By applying state-of-the-art data analytic methods to high-resolution functional magnetic resonance data, researchers in the field have started to record and compare complex patterns of neural activation between brain areas, time points, and conditions. This way, they have gathered tangible evidence that long-term memories of complex objects such as faces and houses are represented as distributed and overlapping patterns of neural activity in the human cortex (e.g., Haxby et al., 2001) and that items which have similar or overlapping neural activation patterns are experienced as similar—be it exemplars of the same category (e.g., Xiao et al., 2016) or items that are falsely remembered as belonging to a memorized set (e.g., Ye et al., 2016). Given this equivalence between the overlap of cortical activation patterns and the experienced psychological overlap of objects in long-term memory, future research should explore whether more complex goal representations which follow the broader definition of a “desired end state” also obey the continuity principle.

To summarize, the work presented here offers an extended view on the principles of goal representations in the context of cognitive control research. Our results suggest that goals can be conceived of as distributed peaks of activation which rise and decay within continuous mental spaces and overlap to varying degrees. Our findings thus support recent theorizing on goal-directed behavior which considers continuity in representational space and time as two core principles that govern the workings in the cognitive system (e.g., Johnson et al., 2009a, 2009b; Lins & Schöner, 2014; Sandamirskaya et al., 2013; Scherbaum et al., 2008; Spivey & Dale, 2004; Wimmer et al., 2014). At the same time, they extend established theories of cognitive control which typically implement goals as single neural units that are largely unconnected from each other (e.g., Botvinick et al., 2001; Cohen et al., 1990; Gilbert & Shallice, 2002; Shenhav et al., 2013; Verguts & Notebaert, 2008). Traditionally, these models have focused on explaining the mechanisms that signal a need for control in an ongoing task as well as the processes that subsequently implement control by adjusting information processing in the cognitive system. While the little emphasis these models put on the representational nature of goals is comprehensible in light of their original purpose, our work highlights the importance to complement such a process-oriented focus with a representation-oriented one. As computational models of cognitive control have fundamentally shaped cognitive psychology, the discrete perspective on goals they implicitly convey has shaped the way we think about goal-directed behavior. This is also reflected in the way goal-directed behavior is studied in the lab where researchers often use standard versions of cognitive control tasks that operate on fixed sets of stimuli and responses, thus thwarting the possibility that the continuity of cognition may manifest in behavior. By applying an adapted, continuous version of a standard set-shifting task, our experiments show that goals can act continuously in representational space. On a broader note, our work highlights the need to become aware of the implicit assumptions and popular beliefs shaping our thinking about and investigations of cognitive control in order to fully understand goal-directed behavior.

## Supplementary Information

Below is the link to the electronic supplementary material.

Supplementary file1 (PDF 786 KB)

## Data Availability

The datasets generated during and analyzed during the current study are available in the Open Science Framework repository (https://osf.io/cg592/).

## Footnotes

1 Since this experiment is the first of its kind, optimal sample sizes could not be estimated a priori. Post hoc analysis of achieved power for the interaction effect using G*Power 3.1 (*N* = 25, Alpha = .05, η_p_^2^ = 0.40) revealed achieved power of 0.998.

2 RT* = RT/Accuracy. According to the procedure in Frisch et al. (2015).

3 For example, 30 participants in Experiment 2 completed 20 trials within each of the cells of the design matrix, which results in 600 trials for each conditions. In contrast, 15 participants in Experiment 3 completed 64 trials within each cell, which results in 960 trials for each condition.

## Code availability

Analysis scripts for all experiments are openly available (https://osf.io/cg592/).

## Declarations

### Open practices statement

Data and analysis scripts for all experiments are openly available online (https://osf.io/cg592/). The experiments were not preregistered. We report how we determined our sample size, all data exclusions (if any), all manipulations, and all measures in the study.
