# Supplementary material for: Continuous goal representations: Distance in representational space affects goal switching
Source: Mem Cognit. 2025 Jan 21;53(6):1656–76. doi: 10.3758/s13421-024-01675-9 (PMC12402036; doi:10.3758/s13421-024-01675-9)
Supplement: Supplementary file 1 — Supplementary file1 (PDF 786 KB) [file 13421_2024_1675_MOESM1_ESM.pdf]

Supplement for

Continuous Goal Representations: Distance in Representational Space Affects Goal Switching

Ulrike Senftleben°, Simon Frisch°, Maja Dshemuchadse†, Stefan Scherbaum° and Caroline Surrey°

° Department of Psychology, Technische Universität Dresden

† Faculty of Social Sciences, Zittau-Görlitz University of Applied Science

Correspondence should be addressed to:

Ulrike Senftleben

Department of Psychology, Technische Universität Dresden

Zellescher Weg 17, 01062 Dresden, Germany

Phone: ++49 351 463 42649

E-mail: ulrike.senftleben@tu-dresden.de

Statistical analyses of response times, error rates, and area under the curve

Here, we present the statistical analyses of response times (RT), error rates (ERR) and the area under the curve (AUC, only experiment 2) for our three experiments. Each analysis is divided into two steps. First, a repeated-measures analysis of variance (RM-ANOVA) is performed on the two-factorial design *Location of Similarity* (Target Similar vs. Distracter Similar) x *Colour Distance* (0° vs. 30° vs. 60° vs 90° in Experiment 1; 0° vs. 20° vs. 40° vs. 60° in Experiment 2; 0° vs. 10° vs. 20° vs. 30° vs. 40° vs. 50° vs. 60° in Experiment 3). If the expected two-way interaction was significant, additional one-way RM-ANOVAs were performed to test the main effects of *Colour Distance* at each of the two levels of the factor *Location of Similarity*. The latter analyses also included the fitting of polynomial contrasts to the data.

Experiment 1

Response Times

In order to test the effect of the factors Location of Similarity and Colour Distance on RT during switch trials of Experiment 1, a 2 (*Location of Similarity*) X 4 (*Colour Distance*) RM-ANOVA was performed. The results are presented in Figure S1 (left) and Table S1.

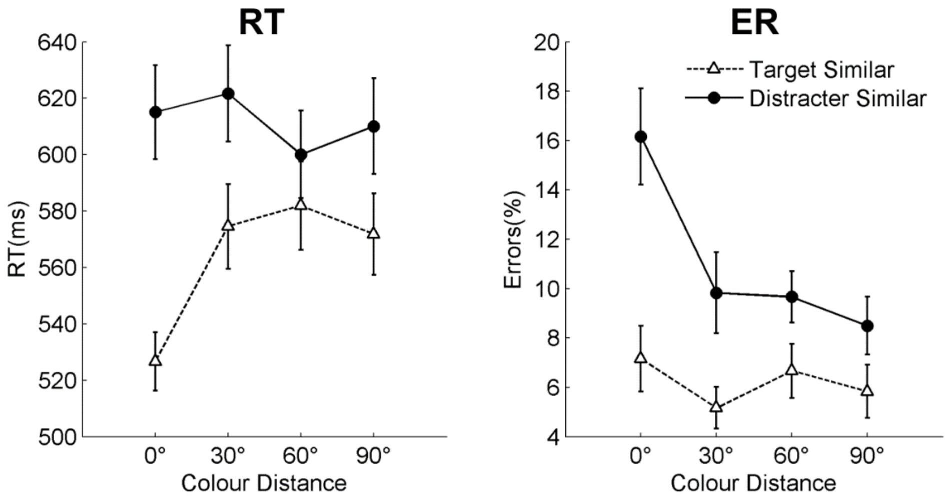

Figure S1. Results of experiment 1 showing response times (left) and error rates (right) during switch trials as a function of *Location of Similarity* and *Colour Distance.* Error bars represent standard errors.

Table S1
RM-ANOVA for effects of the factors Location of Similarity *and* Colour Distance *on RT in switch trials of Experiment 1.*

**Source**	***SC***	***SS***	***df***	***MS***	***F***	***p***
Location of Similarity	None	0.115	1.000	0.115	76.066	<0.001
	GG	0.115	1.000	0.115	76.066	<0.001
Residual	None	0.036	24.000	0.002		
	GG	0.036	24.000	0.002		
Colour Distance	None	0.021	3.000	0.007	7.399	<0.001
	GG	0.021	2.135	0.010	7.399	0.001
Residual	None	0.067	72.000	0.001		
	GG	0.067	51.231	0.001		
Location of Similarity ✻ Colour Distance	None	0.033	3.000	0.011	15.184	<0.001
	GG	0.033	2.552	0.013	15.184	<0.001
Residual	None	0.052	72.000	0.001		
	GG	0.052	61.259	0.001		

Note. GG: Results after Greenhouse-Geisser correction; SC: sphericity correction; SS: sum of squares; MS: mean square.

As the expected interaction of *Location of Similarity* X *Colour Distance* proved statistically reliable, separate RM-ANOVAs were performed to follow up on the effects of *Colour Distance* on RT in both the *Target Similar* and the *Distracter Similar* condition. Results are reported in Table S2.

Table S2
RM-ANOVA for the effects of the factor Switch Distance *on RT in switch trials of the* Target Similar *and the* Distracter Similar *conditions in Experiment 1.*

**Source**	***SC***	***SS***	***df***	***MS***	***F***	***p***
Target Similar						
Colour Distance (CD)	None	0.047	3.000	0.016	22.695	<0.001
	GG	0.047	2.119	0.022	22.695	<0.001
Residual	None	0.050	72.000	0.001		
	GG	0.050	50.857	0.001		
Linear contrast		0.025	1.000	0.025	31.329	<0.001
Quadratic contrast		0.021	1.000	0.021	27.043	<0.001
Cubic contrast		0.001	1.000	0.001	1.356	0.256
Residual (linear)		0.020	24.000	0.001		
Residual (quadratic)		0.019	24.000	0.001		
Residual (cubic)		0.012	24.000	0.000		
Distracter Similar						
Colour Distance	None	0.006	3.000	0.002	2.165	0.100
	GG	0.006	2.462	0.003	2.165	0.113
Residual	None	0.069	72.000	0.001		
	GG	0.069	59.090	0.001		
Linear contrast		0.002	1.000	0.002	1.318	0.262
Quadratic contrast		0.000	1.000	0.000	0.112	0.740
Cubic contrast		0.004	1.000	0.004	4.810	0.038
Residual (linear)		0.030	24.000	0.001		
Residual (quadratic)		0.016	24.000	0.001		
Residual (cubic)		0.022	24.000	0.001		

Note. GG: Results after Greenhouse-Geisser correction; SC: sphericity correction; SS: sum of squares; MS: mean square.

Error Rates

In order to test the effect of the factors *Location of Similarity* and *Colour Distance* on error rates (ER) during switch trials of Experiment 1, a 2 (*Location of Similarity*) X 4 (*Colour Distance*) RM-ANOVA was performed. The results are presented in Figure S1 (right) and Table S3.

Table S3
RM-ANOVA for effects of the factors Location of Similarity *and* Colour Distance *on ER in switch trials of Experiment 1.*

**Source**	***SC***	***SS***	***df***	***MS***	***F***	***p***
Location of Similarity	None	0.117	1.000	0.117	32.640	<0.001
	GG	0.117	1.000	0.117	32.640	<0.001
Residual	None	0.086	24.000	0.004		
	GG	0.086	24.000	0.004		
Colour Distance	None	0.064	3.000	0.021	8.734	<0.001
	GG	0.064	2.790	0.023	8.734	<0.001
Residual	None	0.177	72.000	0.002		
	GG	0.177	66.951	0.003		
Location of Similarity ✻ Colour Distance	None	0.032	3.000	0.011	3.076	0.033
	GG	0.032	2.500	0.013	3.076	0.043
Residual	None	0.248	72.000	0.003		
	GG	0.248	59.988	0.004		

Note. GG: Results after Greenhouse-Geisser correction; SC: sphericity correction; SS: sum of squares; MS: mean square.

As the expected interaction of *Location of Similarity* X *Colour Distance* proved statistically reliable, separate RM-ANOVAs were performed to follow up on the effects of *Colour Distance* on ER in both the *Target Similar* and the *Distracter Similar* condition. Results are reported in Table S4.

Table S4
RM-ANOVA for effects of the factor Switch Distance *on ER in switch trials of the* Target Similar *and the* Distracter Similar *conditions in Experiment 1.*

**Source**	***SC***	***SS***	***df***	***MS***	***F***	***p***
Target Similar						
Colour Distance (CD)	None	0.047	3.000	0.016	22.695	<0.001
	GG	0.047	2.119	0.022	22.695	<0.001
Residual	None	0.050	72.000	0.001		
	GG	0.050	50.857	0.001		
Linear contrast		0.025	1.000	0.025	31.329	<0.001
Quadratic contrast		0.021	1.000	0.021	27.043	<0.001
Cubic contrast		0.001	1.000	0.001	1.356	0.256
Residual (linear)		0.020	24.000	0.001		
Residual (quadratic)		0.019	24.000	0.001		
Residual (cubic)		0.012	24.000	0.000		
Distracter Similar						
Colour Distance	None	0.006	3.000	0.002	2.165	0.100
	GG	0.006	2.462	0.003	2.165	0.113
Residual	None	0.069	72.000	0.001		
	GG	0.069	59.090	0.001		
Linear contrast		0.002	1.000	0.002	1.318	0.262
Quadratic contrast		0.000	1.000	0.000	0.112	0.740
Cubic contrast		0.004	1.000	0.004	4.810	0.038
Residual (linear)		0.030	24.000	0.001		
Residual (quadratic)		0.016	24.000	0.001		
Residual (cubic)		0.022	24.000	0.001		

Note. GG: Results after Greenhouse-Geisser correction; SC: sphericity correction; SS: sum of squares; MS: mean square.

Experiment 2

Response Times

In order to test the effect of the factors *Location of Similarity* and *Colour Distance* on RT during switch trials of Experiment 2, a 2 (*Location of Similarity*) X 4 (*Colour Distance*) RM-ANOVA was performed. The results are presented in Figure S2 (left) and Table S5.

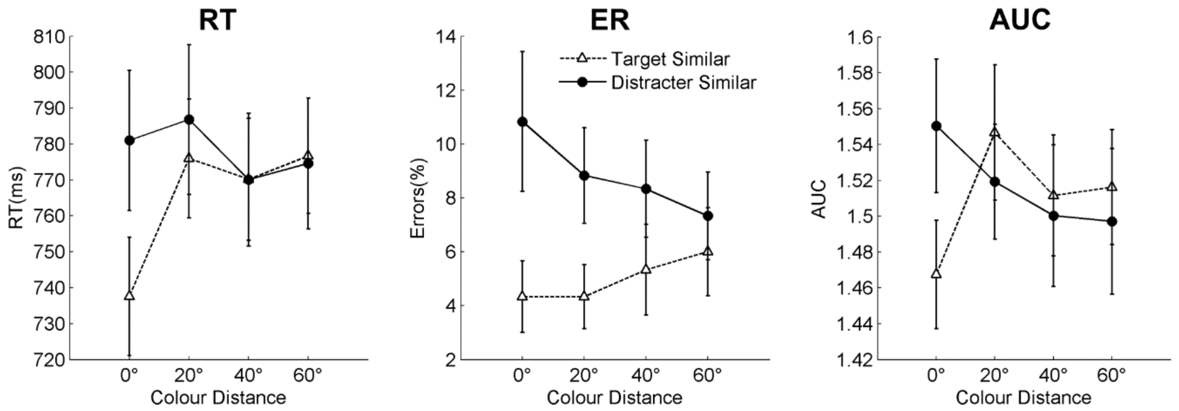

Figure S2. Results of experiment 2 showing response times (left), error rates (middle), and AUCs (right) during switch trials as a function of *Location of Similarity* and *Colour Distance.* Error bars represent standard errors.

Table S5
RM-ANOVA for effects of the factors Location of Similarity *and* Colour Distance *on RT in switch trials of Experiment 2.*

Source	*SC*	*SS*	*df*	*MS*	*F*	*p*
Location of Similarity	None	0.010	1.000	0.010	2.728	0.109
	GG	0.010	1.000	0.010	2.728	0.109
Residual	None	0.108	29.000	0.004		
	GG	0.108	29.000	0.004		
Colour Distance	None	0.016	3.000	0.005	5.091	0.003
	GG	0.016	2.911	0.005	5.091	0.003
Residual	None	0.091	87.000	0.001		
	GG	0.091	84.419	0.001		
Location of Similarity ✻ Colour Distance	None	0.020	3.000	0.007	5.623	0.001
	GG	0.020	2.715	0.007	5.623	0.002
Residual	None	0.103	87.000	0.001		
	GG	0.103	78.737	0.001		

Note. GG: Results after Greenhouse-Geisser correction; SC: sphericity correction; SS: sum of squares; MS: mean square.

As the expected interaction of *Location of Similarity* X *Colour Distance* proved statistically reliable, separate RM-ANOVAs were performed to follow up on the effects of *Colour Distance* on RT in both the *Target Similar* and the *Distracter Similar* condition. Results are reported in Table S6.

Table S6
RM-ANOVA for effects of the factor Switch Distance *on RT in switch trials of the* Target Similar *and the* Distracter Similar *conditions in Experiment 2.*

**Source**	***SC***	***SS***	***df***	***MS***	***F***	***p***
Target Similar						
Colour Distance (CD)	None	0.031	3.000	0.010	9.644	<0.001
	GG	0.031	2.804	0.011	9.644	<0.001
Residual	None	0.093	87.000	0.001		
	GG	0.093	81.318	0.001		
Linear contrast		0.019	1.000	0.019	19.549	<0.001
Quadratic contrast		0.008	1.000	0.008	8.209	0.008
Cubic contrast		0.005	1.000	0.005	3.567	0.069
Residual (linear)		0.028	29.000	0.001		
Residual (quadratic)		0.027	29.000	0.001		
Residual (cubic)		0.039	29.000	0.001		
Distracter Similar						
Colour Distance	None	0.005	3.000	0.002	1.394	0.250
	GG	0.005	2.526	0.002	1.394	0.254
Residual	None	0.100	87.000	0.001		
	GG	0.100	73.250	0.001		
Linear contrast		0.002	1.000	0.002	1.253	0.272
Quadratic contrast		0.000	1.000	0.000	0.011	0.917
Cubic contrast		0.003	1.000	0.003	4.152	0.051
Residual (linear)		0.045	29.000	0.002		
Residual (quadratic)		0.035	29.000	0.001		
Residual (cubic)		0.020	29.000	0.001		

Note. GG: Results after Greenhouse-Geisser correction; SC: sphericity correction; SS: sum of squares; MS: mean square.

Error Rates

In order to test the effect of the factors *Location of Similarity* and *Colour Distance* on ER during switch trials of Experiment 2, a 2 (*Location of Similarity*) X 4 (*Colour Distance*) RM-ANOVA was performed. The results are presented in Figure S2 (middle) and Table S7.

Table S7
RM-ANOVA for effects of the factors Location of Similarity *and* Colour Distance *on ER in switch trials of Experiment 2.*

**Source**	***SC***	***SS***	***df***	***MS***	***F***	***p***
Location of Similarity	None	0.088	1.000	0.088	25.108	<0.001
	GG	0.088	1.000	0.088	25.108	<0.001
Residual	None	0.102	29.000	0.004		
	GG	0.102	29.000	0.004		
Colour Distance	None	0.004	3.000	0.001	0.412	0.745
	GG	0.004	2.521	0.001	0.412	0.710
Residual	None	0.264	87.000	0.003		
	GG	0.264	73.096	0.004		
Location of Similarity ✻ Colour Distance	None	0.022	3.000	0.007	2.188	0.095
	GG	0.022	2.528	0.009	2.188	0.107
Residual	None	0.288	87.000	0.003		
	GG	0.288	73.309	0.004		

Note. GG: Results after Greenhouse-Geisser correction; SC: sphericity correction; SS: sum of squares; MS: mean square.

As the expected interaction of *Location of Similarity* X *Colour Distance* did not prove statistically reliable, no further tests were performed on ER in Experiment 2.

Area under the Curve

In order to test the effect of the factors *Location of Similarity* and *Colour Distance* on AUC during switch trials of Experiment 2, a 2 (*Location of Similarity*) X 4 (*Colour Distance*) RM-ANOVA was performed. The results are presented in Figure S2 (right) and Table S8.

Table S8
RM-ANOVA for effects of the factors Location of Similarity *and* Colour Distance *on AUC in switch trials of Experiment 2.*

Source	*SC*	*SS*	*df*	*MS*	*F*	*p*
Location of Similarity	None	5.7E+05	1.000	5.7E+05	0.299	0.588
	GG	5.7E+05	1.000	5.7E+05	0.299	0.588
Residual	None	5.6E+07	29.000	1.9E+06		
	GG	5.6E+07	29.000	1.9E+06		
Colour Distance	None	3.3E+06	3.000	1.1E+06	0.918	0.436
	GG	3.3E+06	2.551	1.3E+06	0.918	0.424
Residual	None	1.0E+08	87.000	1.2E+06		
	GG	1.0E+08	73.970	1.4E+06		
Location of Similarity ✻ Colour Distance	None	7.1E+06	3.000	2.4E+06	1.990	0.121
	GG	7.1E+06	2.444	2.9E+06	1.990	0.135
Residual	None	1.0E+08	87.000	1.2E+06		
	GG	1.0E+08	70.876	1.5E+06		

Note. GG: Results after Greenhouse-Geisser correction; SC: sphericity correction; SS: sum of squares; MS: mean square.

As the expected interaction of *Location of Similarity* X *Colour Distance* did not prove statistically reliable, no further tests were performed on AUC in Experiment 2.

Experiment 3

Response Times

In order to test the effect of the factors *Location of Similarity* and *Colour Distance* on RT during switch trials of Experiment 3, a 2 (*Location of Similarity*) X 7 (*Colour Distance*) RM-ANOVA was performed. The results are presented in Figure S3 (left) and Table S9.

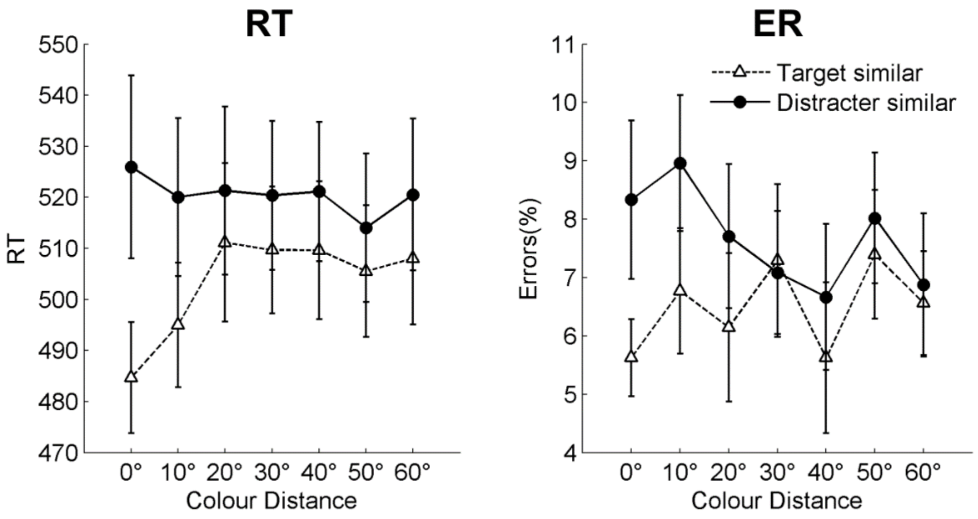

Figure S3. Results of experiment 3 showing response times (left) and error rates (right) during switch trials as a function of *Location of Similarity* and *Colour Distance.* Error bars represent standard errors.

Table S9
RM-ANOVA for effects of the factors Location of Similarity *and* Colour Distance *on RT in switch trials of Experiment 2.*

**Source**	***SC***	***SS***	***df***	***MS***	***F***	***p***
Location of Similarity	None	0.015	1.000	0.015	12.138	0.004
	GG	0.015	1.000	0.015	12.138	0.004
Residual	None	0.018	14.000	0.001		
	GG	0.018	14.000	0.001		
Colour Distance	None	0.003	6.000	0.001	4.973	0.000
	GG	0.003	3.840	0.001	4.973	0.002
Residual	None	0.010	84.000	0.000		
	GG	0.010	53.762	0.000		
Location of Similarity * Colour Distance	None	0.006	6.000	0.001	4.566	0.000
	GG	0.006	3.908	0.002	4.566	0.003
Residual	None	0.020	84.000	0.000		
	GG	0.020	54.706	0.000		

Note. GG: Results after Greenhouse-Geisser correction; SC: sphericity correction; SS: sum of squares; MS: mean square.

As the expected interaction of *Location of Similarity* X *Colour Distance* proved statistically reliable, separate RM-ANOVAs were performed to follow up on the effects of *Colour Distance* on RT in both the *Target Similar* and the *Distracter Similar* condition. Results are reported in Table S10.

Table S10

RM-ANOVA for effects of the factor Switch Distance *on RT in switch trials of the* Target Similar *and the* Distracter Similar *conditions in Experiment 3.*

**Source**	***SC***	***SS***	***df***	***MS***	***F***	***p***
Target Similar						
Colour Distance (CD)	None	0.009	6.000	0.001	7.449	<0.001
	GG	0.009	3.479	0.003	7.449	<0.001
Residual	None	0.017	84.000	0.000		
	GG	0.017	48.704	0.000		
Linear contrast		0.004	1.000	0.004	18.281	0.001
Quadratic contrast		0.003	1.000	0.003	16.731	0.001
Cubic contrast		0.001	1.000	0.001	2.867	0.113
Fourth-order contrast		0.000	1.000	0.000	2.219	0.159
Fifth-order contrast		0.000	1.000	0.000	0.648	0.434
Sixth-order contrast		0.000	1.000	0.000	0.755	0.400
Residual (linear)		0.003	14.000	0.000		
Residual (quadratic)		0.003	14.000	0.000		
Residual (cubic)		0.002	14.000	0.000		
Residual (fourth-order)		0.002	14.000	0.000		
Residual (fifth-order)		0.003	14.000	0.000		
Residual (sixth-order)		0.003	14.000	0.000		
Distracter Similar						
Colour Distance	None	0.001	6.000	0.000	1.179	0.326
	GG	0.001	4.206	0.000	1.179	0.330
Residual	None	0.013	84.000	0.000		
	GG	0.013	58.886	0.000		
Linear contrast		0.000	1.000	0.000	1.594	0.227
Quadratic contrast		0.000	1.000	0.000	0.648	0.434
Cubic contrast		0.000	1.000	0.000	0.011	0.919
Fourth-order contrast		0.000	1.000	0.000	2.890	0.111
Fifth-order contrast		0.000	1.000	0.000	0.530	0.479
Sixth-order contrast		0.000	1.000	0.000	0.742	0.404
Residual (linear)		0.004	14.000	0.000		
Residual (quadratic)		0.002	14.000	0.000		
Residual (cubic)		0.002	14.000	0.000		
Residual (fourth-order)		0.002	14.000	0.000		
Residual (fifth-order)		0.001	14.000	0.000		
Residual (sixth-order)		0.002	14.000	0.000		

Note. GG: Results after Greenhouse-Geisser correction; SC: sphericity correction; SS: sum of squares; MS: mean square.

Error Rates

In order to test the effect of the factors *Location of Similarity* and *Colour Distance* on ER during switch trials of Experiment 3, a 2 (*Location of Similarity*) X 7 (*Colour Distance*) RM-ANOVA was performed. The results are presented in Figure S3 (right) and Table S11.

Table S11
RM-ANOVA for effects of the factors Location of Similarity *and* Colour Distance *on ER in switch trials of Experiment 2.*

**Source**	***SC***	***SS***	***df***	***MS***	***F***	***p***
Location of Similarity	None	0.000	1.000	0.000	0.081	0.780
	GG	0.000	1.000	0.000	0.081	0.780
Residual	None	0.007	14.000	0.001		
	GG	0.007	14.000	0.001		
Colour Distance	None	0.001	6.000	0.000	0.185	0.980
	GG	0.001	3.853	0.000	0.185	0.941
Residual	None	0.080	84.000	0.001		
	GG	0.080	53.948	0.001		
Location of Similarity ✻ Colour Distance	None	0.005	6.000	0.001	1.008	0.426
	GG	0.005	3.259	0.001	1.008	0.403
Residual	None	0.067	84.000	0.001		
	GG	0.067	45.623	0.001		

Note. GG: Results after Greenhouse-Geisser correction; SC: sphericity correction; SS: sum of squares; MS: mean square.

As the expected interaction of *Location of Similarity* X *Colour Distance* did not prove statistically reliable, no further tests were performed on ER in Experiment 3.

Statistical analyses of the full research design

Here, we present the statistical analyses of the full research design of experiments 1 to 3. The full designs contained both levels of the experimental factor *Repetition*, (i.e. trials preceding a switch [preswitch trials] vs. switch trials) in addition to the two other experimental factors *Location of Similarity* (Target Switches [TS] vs. Distracter Switches [DS]), and *Colour Distance* (0° vs. 30° vs. 60° vs. 90° in experiment 1; 0° vs. 20° vs. 40° vs. 60° in experiment 2; 0° vs. 10° vs. 20° vs. 30° vs. 40° vs. 50° vs 60° in experiment 3).

Experiment 1

We ran a three-factorial repeated-measures analysis of variance (RM-ANOVA) including *Repetition*, *Location of Similarity* and *Colour Distance* as independent variables and RT* as dependent variable. We found significant main effects for the factors *Repetition*, $F(1, 24) = 97.96$, $p < .001$, $\eta_p^2 = 0.80$, and *Location of Similarity*, $F(1, 24) = 55.27$, $p < .001$, $\eta_p^2 = 0.70$, indicating that, overall, performance was better in preswitch as compared to switch trials and better in TS than in DS trials (see *Figure S4*). In addition to two significant two-way interactions *Repetition* x *Location of Similarity*, $F(1, 24) = 73.39$, $p < .001$, $\eta_p^2 = 0.75$ and *Colour Distance* x *Location of Similarity*, $F(3, 72) = 8{:}49$, $p < .001$, $\eta_p^2 = 0.26$, the three-way interaction *Repetition* x *Location of Similarity* x *Colour Distance* was significant as well, $F(3, 72) = 14.33$, $p < .001$, $\eta_p^2 = 0.37$. This indicated that, as predicted, performance depended on the combined effect of *Location of Similarity* and *Colour Distance* and that the size of this combined effect varied between preswitch and switch trials. To follow up on this effect, we computed two additional RM-ANOVAs for preswitch and switch trials separately. For preswitch trials, this analysis revealed no significant effects (all *p*s > .05; all *F*s < 4.12) indicating that, at the end of the run (i.e. during preswitch trials), the effects of the experimental manipulations *Location of Similarity* and *Colour Distance* had worn off. In contrast, in switch trials, we found the predicted two-way interaction of *Location of Similarity*

and *Colour Distance, F*(3, 72) = 15.79, p < .001, η_p^2 = 0.40. This shows that participant's ability to attend to or ignore a source of information during switch trials does depend on how similar this information is to the goal pursued before. For further details on this analysis, please see the main body of the text.

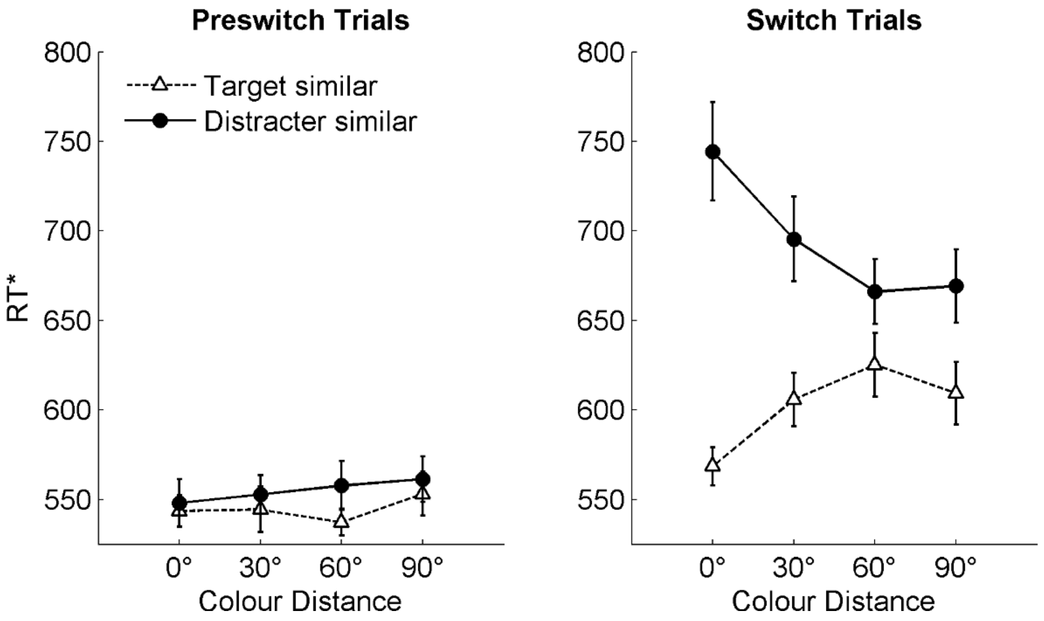

*Figure S4.*Results of experiment 1 showing the inverse efficiency score RT* as a function of *Location of Similarity* and *Colour Distance* for trials preceding a switch (left) and switch trials (right), separately. Error bars represent standard errors.

Experiment 2

RT.* The three-factorial RM-ANOVA revealed significant main effects for the factors *Repetition, F*(1, 29) = 63.21, p < .001, η_p^2 = 0.70, and *Location of Similarity, F*(1, 29) = 23.92, p < .001, η_p^2 = 0.45, indicating that, overall, performance was better in preswitch as compared to switch trials as well as in TS as compared to DS trials (see Figure S5). In addition to the significant two-way interactions *Repetition* x *Location of Similarity, F*(1, 29) = 5-47, p = .03, η_p^2 = 0.16 and *Location of Similiarty* x *Colour Distance, F*(3, 87) = 3.91, p = .02, η_p^2 = 0.12, the expected three-way interaction *Repetition x Location of Similarity x Colour Distance* proved to be statistically reliable as well, *F*(3, 87) = 4.37, p = .02, η_p^2 = 0.13. To follow up on this effect, we computed two additional RM-ANOVAs

for preswitch and switch trials separately. For preswitch trials, this analysis only revealed a significant main effect for the factor *Location of Similarity*, $F(1, 29) = 6.14$, $p = .02$, $\eta_p^2 = 0.18$, indicating that performance during preswitch trials in the DS condition was overall worse than performance during preswitch trials in the TS condition. All other effects were non-significant (all *F*s < 1:83; all *p*s > .15). In contrast, in switch trials, we found the predicted two-way interaction of *Location of Similarity* and *Colour Distance*, $F(3, 87) = 5.85$, $p < .01$, $\eta_p^2 = 0.17$, indicating that the combined effects of both factors shaped participants ability to respond during switch trials. For further details on this analysis, please see the main body of the text.

*AUC**. The three-factorial RM-ANOVA revealed significant main effects for the factors *Repetition*, $F(1, 29) = 26.27$, $p < .001$, $\eta_p^2 = 0.48$, and *Location of Similarity*, $F(1, 29) = 19.68$, $p < .001$, $\eta_p^2 = 0.40$, indicating that, overall, performance was better in preswitch as compared to switch trials as well as in TS as compared to DS trials (see Figure S6). While none of the two-way interactions reached significance (all *F*s < 3.60, all *p*s > .06), the three-way interaction *Repetition x Location of Similarity x Colour Distance* was statistically reliable, $F(3, 87) = 3.55$, $p = .03$, $\eta_p^2 = 0.11$. To follow up on this effect, we computed two additional RM-ANOVAs for preswitch and switch trials separately. For preswitch trials, this analysis only revealed a significant main effect for the factor *Colour Distance*, $F(3, 87) = 3.43$, $p = .04$, $\eta_p^2 = 0.11$. However, Bonferroni-corrected pairwise comparisons that were calculated to follow up on this effect showed no significant differences in AUC* between specific levels of the *Colour Distance* manipulation (all *p*s > .15). In contrast, the RM-ANOVA for switch trials not only showed a significant main effect of *Location of Similarity*, $F(1, 29) = 18.30$, $p < .001$, $\eta_p^2 = 0.39$, but also the predicted interaction of *Location of Similarity* and *Colour Distance*, $F(3, 87) = 3.70$, $p = .03$, $\eta_p^2 = 0.11$. For further details on this analysis, please see the main body of the text.

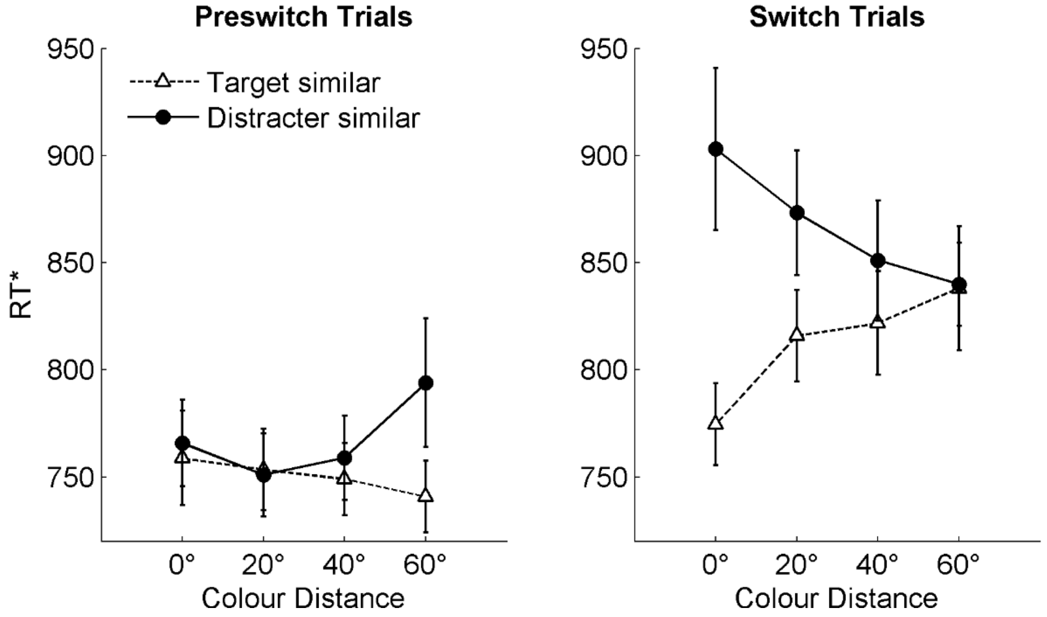

Figure S5. Results of experiment 2 showing the inverse efficiency score RT* as a function of Location of Similarity and Colour Distance for trials preceding a switch (left) and switch trials (right), separately. Error bars represent standard errors.

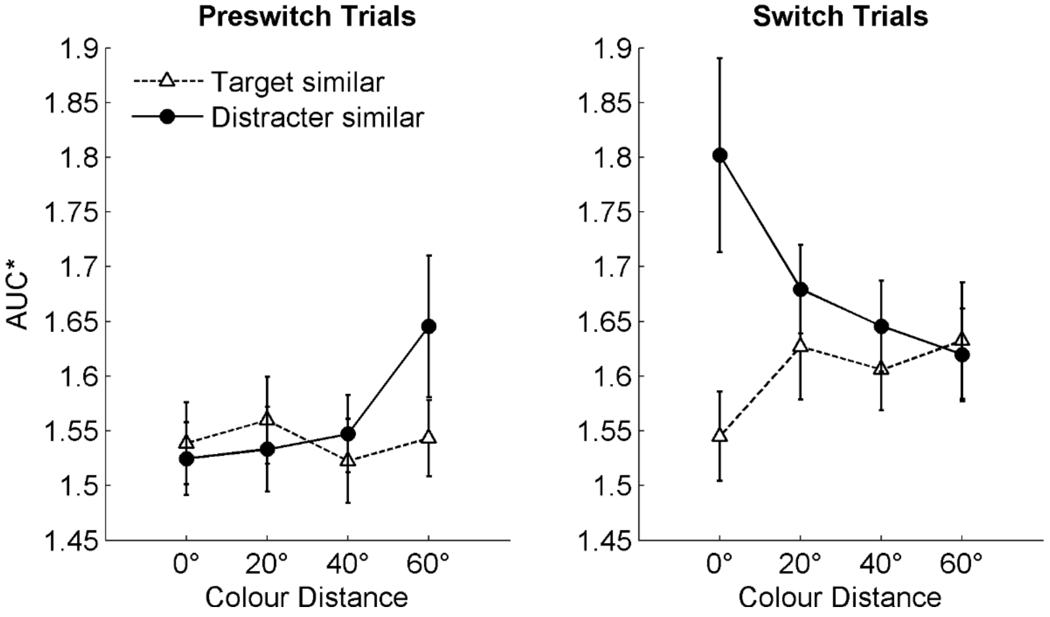

Figure S6. Results of experiment 2 showing AUC* as a function of Location of Similarity and Colour Distance for trials preceding a switch (left) and switch trials (right), separately. Error bars represent standard errors.

Experiment 3

Again, the three-factorial RM-ANOVA revealed significant main effects for the factors *Repetition*, $F(1, 14) = 66.55$, $p < .001$, $\eta_p^2 = 0.83$, and *Location of Similarity*,

F(1, 14) = 8.74, p = .01, η_p^2 = 0.38, indicating that, overall, participants performed worse in switch as compared to repetition trials and worse in DS as compared to TS trials (see Figure S7). Apart from the significant two-way interactions *Repetition* x *Location of Similarity*, F(1, 14) = 8.35, p = .012, η_p^2 = 0.37, and *Colour Distance* x *Location of Similarity*, F(6, 84) = 2.35, p = .04, η_p^2 = 0.14, the predicted three-way interaction *Repetition* x *Location of Similarity* x *Colour Distance* was significant as well, F(6, 84) = 3.22, p < .01, η_p^2 = 0.19, indicating that *Location of Similarity* and *Colour Distance* had a combined effect on performance that differed between preswitch and switch trials. To follow-up on this effect, we calculated one RM-ANOVA for each level of the factor *Repetition*. For preswitch trials, this analysis revealed no significant effects (all ps > .33, all Fs < 1.17), indicating that performance was similar across all conditions at the end of a run. In contrast, for switch trials we found the expected interaction of *Location of Similarity* and *Colour Distance*, F(6, 84) = 3.59, p < .01, η_p^2 = 0.20. For further details on this analysis, please see the main body of the text.

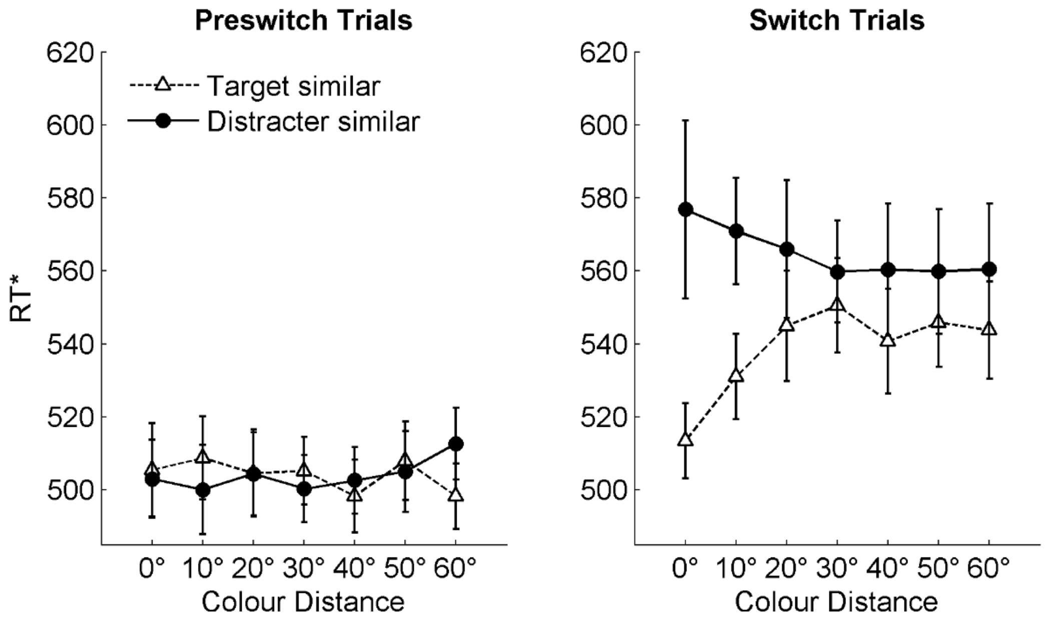

Figure S7. Results of experiment 3 showing the inverse efficiency score RT* as a function of Location of Similarity and Colour Distance for trials preceding a switch (left) and switch trials (right), separately. Error bars represent standard errors.

Practice and Fatigue effects

To assess potential practice or fatigue effects, we repeated our analyses of RT* separately for the data from the first two sessions and from the last two sessions (see Figure S8). For the first two sessions, all results were qualitatively the same as described in the main text. For the last two sessions, only the main effect of Location of Similarity remained significant, $F(1,14) = 5.15$, $p = .04$, $\eta_p^2 = 0.269$. The interaction effect was no longer significant, $F(1,14) = 1.50$, $p = .187$, $\eta_p^2 = 0.097$.

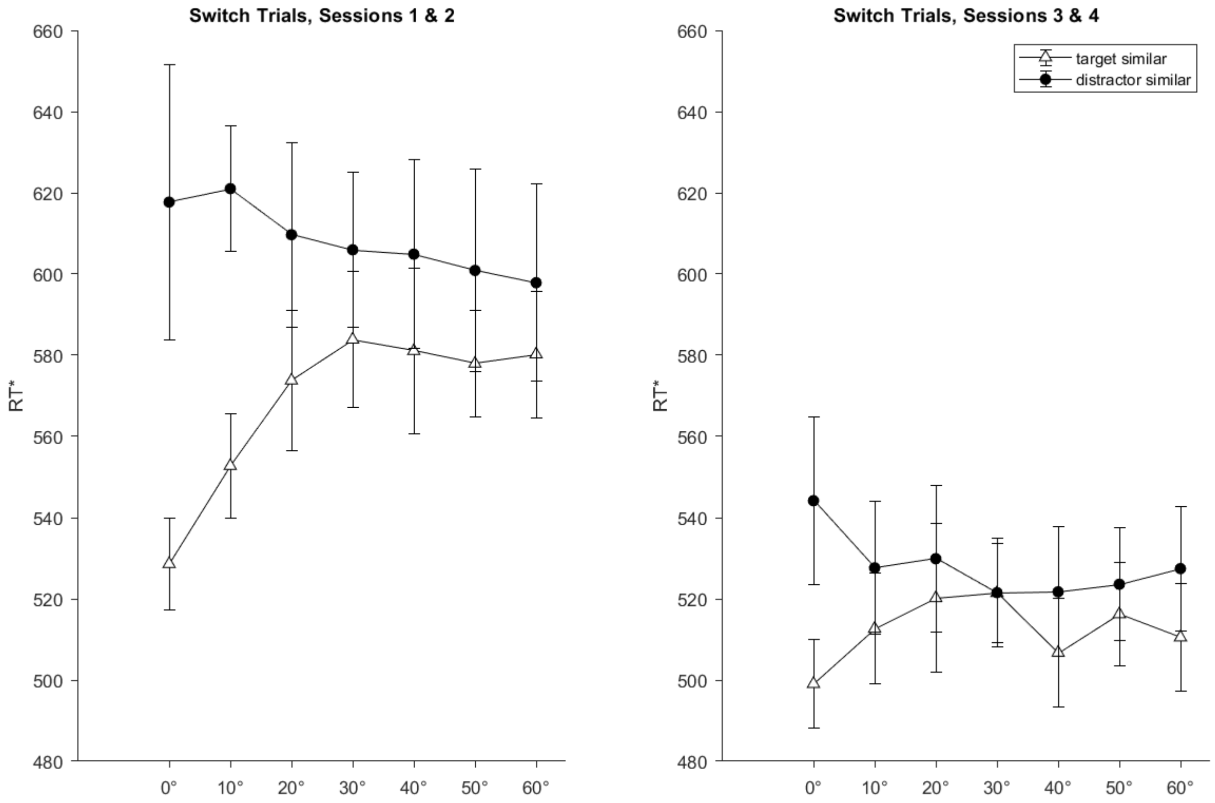

Figure S8: RT* for target and distractors separately for the first two and the last two sessions.
